# Growth periodicity in semi‐deciduous tropical tree species from the Congo Basin

**DOI:** 10.1002/pei3.10144

**Published:** 2024-05-22

**Authors:** Basile Luse Belanganayi, Claire Delvaux, Elizabeth Kearsley, Kévin Lievens, Mélissa Rousseau, Christophe Mbungu Phaka, Brice Yannick Djiofack, Félix Laurent, Nils Bourland, Wannes Hubau, Tom De Mil, Hans Beeckman

**Affiliations:** ^1^ Forest is Life, TERRA Teaching and Research Centre, Gembloux Agro‐Bio Tech University of Liège Gembloux Belgium; ^2^ Service of Wood Biology Royal Museum for Central Africa (RMCA) Tervuren Belgium; ^3^ Woodwise Brussels Belgium; ^4^ BlueGreen Labs Melsele Belgium; ^5^ Institut National Pour l'Etudes et la Recherche Agronomiques Kinshasa Democratic Republic of the Congo; ^6^ Department of Forest and Water Management Gent University Ghent Belgium

**Keywords:** cambial marking, growth‐ring distinctness, periodicity of growth‐ring formation, secondary growth, tropical forests

## Abstract

In the tropics, more precisely in equatorial dense rainforest, xylogenesis is driven by a little distinct climatological seasonality, and many tropical trees do not show clear growth rings. This makes retrospective analyses and modeling of future tree performance difficult. This research investigates the presence, the distinctness, and the periodicity of growth ring for dominant tree species in two semi‐deciduous rainforests, which contrast in terms of precipitation dynamics. Eighteen tree species common to both forests were investigated. We used the cambial marking technique and then verified the presence and periodicity of growth‐ring boundaries in the wood produced between pinning and collection by microscopic and macroscopic observation. The study showed that all eighteen species can form visible growth rings in both sites. However, the periodicity of ring formation varied significantly within and between species, and within sites. Trees from the site with clearly defined dry season had a higher likelihood to form periodical growth rings compared to those from the site where rainfall seasonality is less pronounced. The distinctness of the formed rings however did not show a site dependency. Periodical growth‐ring formation was more likely in fast‐growing trees. Furthermore, improvements can be made by a detailed study of the cambial activity through microcores taken at high temporal resolution, to get insight on the phenology of the lateral meristem.

## Graphical abstract

In the tropical Congo Basin, our study revealed varying tree‐ring formation among species and sites. While all monitored trees exhibited growth rings, their periodicity differed significantly. Notably, the distinctness of the rings was consistent across sites, and faster‐growing trees exhibited a higher likelihood of periodic ring formation.

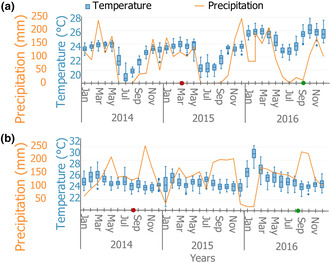

## 1 INTRODUCTION

Trees archive information on secondary growth dynamics in their xylem (Babst et al., 2014). They form growth rings when they experience cambial dormancy related to adverse conditions (Brienen et al., 2016) including the severity and duration of the dry seasons (Worbes, 1999), flooding in floodplain forests (Schöngart et al., 2002), and salinity fluctuations in mangrove forests (Chowdhury et al., 2008). Analysis of ring patterns on pith‐to‐bark samples makes it possible to estimate the age of trees, to evaluate past reactions to environmental fluctuations, and possibly to reconstruct past climatic conditions (Anchukaitis, 2017). However, temporal morphological markers, such as anatomically distinct growth‐ring boundaries, are irregular or difficult to detect in many tropical trees, making retrospective analyses difficult in tropical forests (Tarelkin et al., 2016).

Recent studies conducted in tropical forests have challenged the paradigm that tropical tree species do not form growth rings (López et al., 2012; Mariaux, 2016). Despite the great diversity in appearance and occurrence of growth rings in tropical regions, their annual character has been increasingly identified in more and more tree species (Worbes, 2002, 2011; Zuidema et al., 2012). Despite these recent advances, the forests of the Congo Basin are still under‐represented in studies highlighting ring formation by tree species (Couralet et al., 2013). Particularly, it remains unclear whether there is no cambial dormancy, or a single or multiple dormancy period(s) during a growing season.

Given the strong link between carbon sequestration and tree growth (Babst et al., 2014), it is also of key importance to assess the periodicity of secondary growth and understand its drivers, in order to forecast the feedback of forests to climate change and the carbon dynamics of the atmosphere (Lehnebach et al., 2021).

In the present study, we investigated growth‐ring presence, distinctness, and periodicity in eighteen tree species growing under two different climate conditions in the Congo Basin (Kottek et al., 2006). As such, wood samples were analyzed in the semi‐deciduous rainforests of the Biosphere Reserves of Luki and Yangambi, in the Democratic Republic of Congo to address the research questions: (1) Do a majority of tree species produce annual and anatomically distinct growth rings? (2) Is growth ring regular between individuals of the same tree species, and what could cause irregularity? (3) What is the effect of site on the periodicity of growth‐ring formation?

Ring boundary can be examined by naked eye or microscopically (Wheeler & Baas, 1998; Brienen & Zuidema, 2005; Chowdhury et al., 2016). The periodicity of wood anatomical traits refers to the regularity/frequency of their appearance over time. Growth rings are considered distinct, when an abrupt structural change at the boundaries between them is present, usually including a change in fiber wall thickness and/or fiber radial diameter. The abrupt changes should enable each cell of the ring boundary to be associated exactly with a single growth ring of the two concomitant ones (Tarelkin et al., 2016). Indistinct growth rings are those that are vague and marked by gradual structural changes at their poorly defined or non‐visible boundaries (IAWA Committee, 1989). The observation of distinct or indistinct rings implies a wood anatomical assessment. This is most often done at high microscopic resolution on a small fragment along the tree circumference. A distinct or an indistinct border can be a very local phenomenon in a tree stem. Wedging rings, which occur very often in tropical wood, are known to hamper considerably the classical growth‐ring analysis. Ring borders can also be of aperiodic nature. Fundamental of growth‐ring analysis, certainly in the tropics, is information on their distinctness and their periodicity (Tarelkin et al., 2016).

We hypothesize differences in growth‐ring formation between the two sites related to differences in rainfall seasonality and the severity of the dry seasons, and between different species related to inherent growth traits. More specifically, we hypothesize (1) growth rings to be predominantly annual in Luki as opposed to Yangambi where the dry season is less pronounced; (2) deciduous species to produce annual growth rings in both sites; and (3) evergreen species to produce annual growth rings only in Luki where the rainfall seasonality is more pronounced.

## 2 MATERIALS AND METHODS

### 2.1 Study sites

**FIGURE 1:**
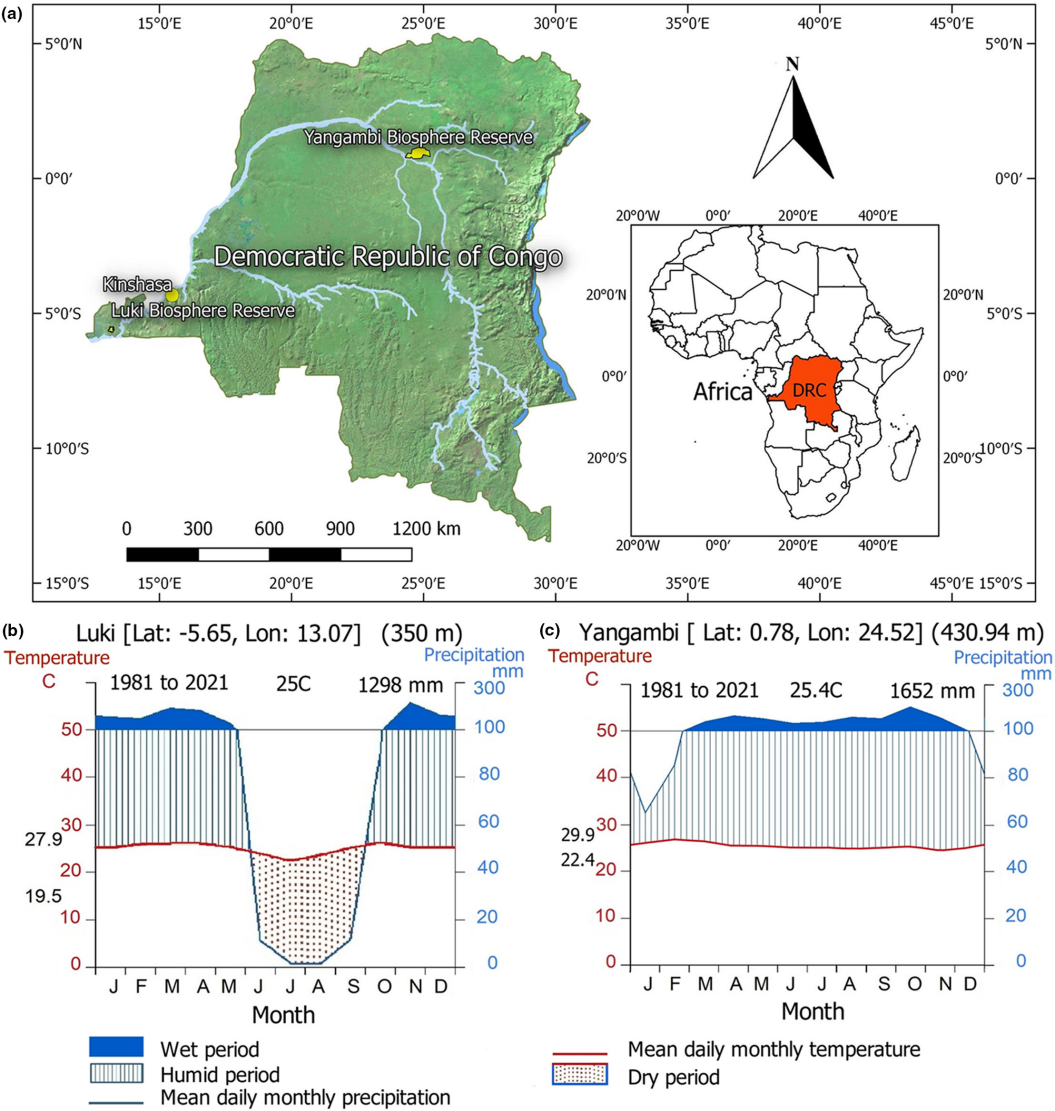
Location of study sites in the Democratic Republic of Congo (a). Walter‐Lieth Climate Diagram of Luki (b) and Yangambi (c) UNESCO Man and Biosphere Reserve from 1981 to 2021. Data were obtained from https://power.larc.nasa.gov/data‐access‐viewer/. The blue line indicates the precipitation curve, the red line indicates the temperature curve, and the dry season is shown in dashed red. The blue stripes indicate the humid period, and the blue area shows the wet period. Temperatures in black, on the left axis, represent the average minimum temperature of the coldest month and the average maximum temperature of the warmest month, respectively. The annual average temperature and annual precipitation are shown in the upper right corner of the diagram.

Study sites are located in the semi‐deciduous rainforests of the Biosphere Reserves of Luki and Yangambi in the Democratic Republic of Congo (Figure 1a). The two sites, while in different areas of the Congo Basin, have many tree species in common. The sites are characterized by a different climate: Luki has an Aw climate and Yangambi an Af climate according to the Köppen classification updated by Kottek et al. (2006). The average annual rainfall is 1296 mm in Luki and 1652 mm in Yangambi, while the average annual temperatures are 25.0°C (ranging from 19.5 to 27.9°C) and 25.4°C (ranging from 22.4 to 29.9°C), respectively (deduced from data from https://power.larc.nasa.gov/data‐access‐viewer/). The dry period extends from June to September in Luki (monthly rainfall <50 mm), while there is no clear dry period in Yangambi, except that there is a decrease in rainfall of about 60 mm per month between December and February (Figure 1a,c).

### 2.2 Study species

We selected 18 tree species (belonging to 13 families) that occur on both study sites. Three of the species are classified as deciduous, and 15 as evergreen. Species common to both the canopy and understory are included (Table 1). This classification of leaf phenological and stratum classes was based on literature (Lubini, 1997), and classes are used as such in further analysis.

**TABLE 1:** Leaf functional group, tree species, botanical family, stratum at adult stage (Lubini, 1997), distinctness and description of growth‐ring boundaries (obtained from this study), and total number of successfully marked trees per species, in Luki and Yangambi forests.

Leaf functional group	Species	Family	Stratum	Distinctness of growth‐ring boundaries	Description of growth‐ring boundaries	No. of trees per site	
						Luki	Yangambi
Deciduous	*Erythrophleum suaveolens* (Guill. & Perr.) Brenan	Caesalpiniaceae	Canopy	Distinct or indistinct	Flattened fibers Band of marginal parenchyma	2	2
	*Petersianthus macrocarpus* (P.Beauv.) Liben	Lecythidaceae	Canopy	Distinct or indistinct	Flattened fibers Band of marginal parenchyma Tangential band with little or no apotracheal axial parenchyma	15	11
	*Prioria oxyphylla* (Harms) Breteler	Caesalpiniaceae	Canopy	Distinct	Flattened fibers Band of marginal parenchyma	2	2
Evergreen	*Blighia welwitschii* (Hiern) Radlk	Sapindaceae	Canopy	Distinct or indistinct	Flattened fibers	2	7
	*Carapa procera* DC	Meliaceae	Understory	Distinct	Band of marginal parenchyma	4	1
	*Celtis mildbraedii* Engler	Cannabaceae	Canopy	Distinct or indistinct	Flattened fibers	12	5
	*Chrysophyllum africanum* A. DC	Sapotaceae	Canopy	Distinct or indistinct	Flattened fibers	8	5
	*Cola griseiflora* De Wild	Malvaceae	Understory	Distinct or indistinct	Tangential band with little or no apotracheal axial parenchyma	6	1
	*Garcinia punctata* Oliver	Clusiaceae	Understory	Distinct or indistinct	Tangential band with little or no apotracheal axial parenchyma	2	3
	*Greenwayodendron suaveolens* (Engl. & Diels) Verdc	Annonaceae	Understory	Distinct or indistinct	Flattened fibers	7	6
	*Leplaea thompsonii* Sprague & Hutch	Meliaceae	Canopy	Distinct or indistinct	Flattened fibers	3	4
	*Pentaclethra macrophylla* Bentham	Fabaceae	Canopy	Distinct or indistinct	Flattened fibers Tangential band without vessels or axial parenchyma	5	1
	*Pycnanthus angolensis* (Welw.) Warb.	Myristicaceae	Canopy	Distinct or indistinct	Flattened fibers	7	1
	*Staudtia kamerunensis* Warb	Myristicaceae	Canopy	Distinct or indistinct	Flattened fibers Band of marginal parenchyma	2	5
	*Strombosiopsis tetrandra* Engler	Olacaceae	Canopy	Distinct or indistinct	Flattened fibers	6	5
	*Trichilia gilgiana* Harms	Meliaceae	Understory	Distinct or indistinct	Flattened fibers Band of marginal parenchyma	12	1
	*Trichilia prieuriana* Juss	Meliaceae	Understory	Distinct or indistinct	Flattened fibers Band of marginal parenchyma	17	6
	*Trilepisium madagascariense* DC	Moraceae	Canopy	Distinct or indistinct	Flattened fibers Band of marginal parenchyma	13	10
Total						125	76

### 2.3 Sample collection

Cambial marking was performed on 3 to 20 individuals per species and per site, depending on availability, a total of 317 trees. On each selected tree, the cambial zone was wounded with a pin at 130 cm height, according to the cambium marking technique recommended by Mariaux (2016), from March 3 to March 7, 2015, in Luki, and from August 4 to August 8, 2014, in Yangambi. For each individual tree, the diameter at breast height (DBH, 130 cm height) was measured with a diameter tape.

The collection of cores with a diameter of 5 cm containing the pinned zone was done from August 29 to September 10, 2016, in Luki and from August 8 to August 18, 2016, in Yangambi. At this time of sample collection, the DBH of each individual tree was remeasured, providing the macroscopic increment during the observation period.

Collected samples were transferred to the wood biology laboratory of the Royal Museum for Central Africa (RMCA) in Tervuren, Belgium, where they were air‐dried, given a Tervuren Wood (Tw) collection accession number, and placed in the wood collection (Figure S1). As such, wood samples were available with two exactly known dates of cambium positions: the time of pinning, revealed by a pin mark, and the time of sampling, revealed by the wood‐bark boundary.

### 2.4 Sample processing and analysis

All samples were sanded on the pinned cross‐sectional plan to highlight the pin mark. Marking was successful on only 201 trees (125 from Luki and 76 from Yangambi, Table 1), and these were used for further analysis. Successful marking means that the insertion of the pin through the cambial zone had left a scar on xylem and caused a production of wound tissue on the wood formed later, near the pinning point (Seo et al., 2007). Wood anatomical observation and analysis were carried out by three observers, with (1) a SZH10 Olympus stereomicroscope equipped with a UC30 Olympus camera connected to a computer, and (2) an HRX‐01 3D Digital Microscope (Figure S2). This also allowed us to capture high‐definition images to examine the presence and distinctness of growth‐ring boundaries, and to measure the wood produced between pinning and collection. The types of software Cell Beta and Hirox were used for image capture. We measured the radial growth, outside the reaction zone, to the left and right of the pinning point using the ImageJ software (Schneider et al., 2012). The mean value, in millimeters, was taken as the radial increase of the tree (Robert et al., 2011). The position of the pin mark in the xylem and the number of growth rings formed after pinning date were determined, and the periodicity (sub‐annual, annual, or no periodicity) was derived.

In accordance with the terminology of the IAWA, which considers a growth‐ring distinct when characterized by boundaries defined by an abrupt structural change usually including a change in fiber wall thickness and/or fiber radial diameter (IAWA Committee, 1989), we considered a growth‐ring distinct when all three observers could associate each cell of the growth‐ring boundary with exactly one of the two concomitant growth rings (Tarelkin et al., 2016). A growth ring is indistinct when at high magnification, it is not possible to associate a single cell to a particular growth ring. Growth‐ring production was considered periodic when the tree produces a constant number of rings per year.

### 2.5 Auxiliary leaf phenological data

In order to verify the general relationship between deciduousness and presence of growth rings, historical long‐term phenological observation records from Luki (Couralet et al., 2013; data from 1948 to 1957) and Yangambi (Hufkens & Kearsley, 2023; data from 1937 to 1956) were assessed. These two datasets contain historical ground‐based leaf phenological observations of local tropical trees made three or four times each month, at Luki and Yangambi, respectively, by the forestry division of the INEAC (Institut National pour l'Étude Agronomique du Congo). The timing at which the canopy contained no (or very few) leaves or was in a state of leaf turnover (partial leaf loss and flushing of new leaves, while retaining significant canopy leaf biomass) was recorded. The average species‐level duration of these phenophase events and the presence of (sub‐)annual periodicity were determined. Periodicity in the phenological timeseries was determined using a Fourier analysis following the method detailed in Bush et al. (2017). More details on the Fourier analysis as applied to these datasets can be found in Kearsley et al. (2024). Four species under investigation (*Garcinia punctata*, *Cola griseiflora*, *Leplaea thompsonii,* and *Strombosiopsis tetrandra*) were not available in the Luki dataset. Genus‐level assessments were made for *Garcinia* sp., *Cola* sp., and *Leplaea* sp. The genus *Strombosiopsis* was not present in the Luki dataset.

### 2.6 Data analysis

To assess the distinctness and the periodicity of growth ring of individual trees as dependent on their inherent species‐specific traits and growing conditions, we fitted a generalized linear mixed‐effects model (GLMM) with a binomial link function using the R (R Core Team, 2023) ‘glmmTMB’ package (Brooks et al., 2017). Binomial for the periodicity of growth‐ring formation was as follows: no periodicity = 0, annual and sub‐annual periodicity = 1, and for growth‐ring distinctness: indistinct = 0, distinct = 1.

The fixed effects of individual tree‐level parameters DBH and relative mean increment are assessed, as are the effects of species‐specific classifications of stratum (understory, canopy) and leaf phenology (evergreen, deciduous), and species‐level derived features of average leaf phenophase duration (state of no leaves, state of leaf turnover; duration in weeks) and the periodicity of these phenophase events ((sub)‐annual or no annual periodicity). The site (Luki, Yangambi) at which the tree was observed is included as a fixed effect. The tree species nested within site is included as a random effect to account for species‐specific effects at site level. The full GLMM models assessed were as follows:

1. periodicity (binary) ~ DBH + Relative mean increment + Stratum + Leaf phenology + Mean leaf phenophase duration + Leaf phenophase periodicity + Site + (1|Site/Species).
2. distinctness (binary) ~ DBH + Relative mean increment + Stratum + Leaf phenology + Mean leaf phenophase duration + Leaf phenophase periodicity + Site + (1|Site/Species).

The inclusion of each fixed effect is assessed (i.e., all combinations of fixed effects), and the best model fit was selected by comparing Akaike Information Criterion (AIC; Burnham & Anderson, 2002) and the standard deviation of residuals $\begin{array}{c} \left(\text{residual},\;,\text{standard},\;,\text{error;},\;,\mathrm{SE},=,\sqrt{\frac{1}{\mathrm{df}}\sum_{i=1}^{n}{\left(Y_{i}-{\hat{Y}}_{i}\right)}^{2}}\right) \end{array}$.

## 3 RESULTS

### 3.1 Distinctness of growth ring

**FIGURE 2:**
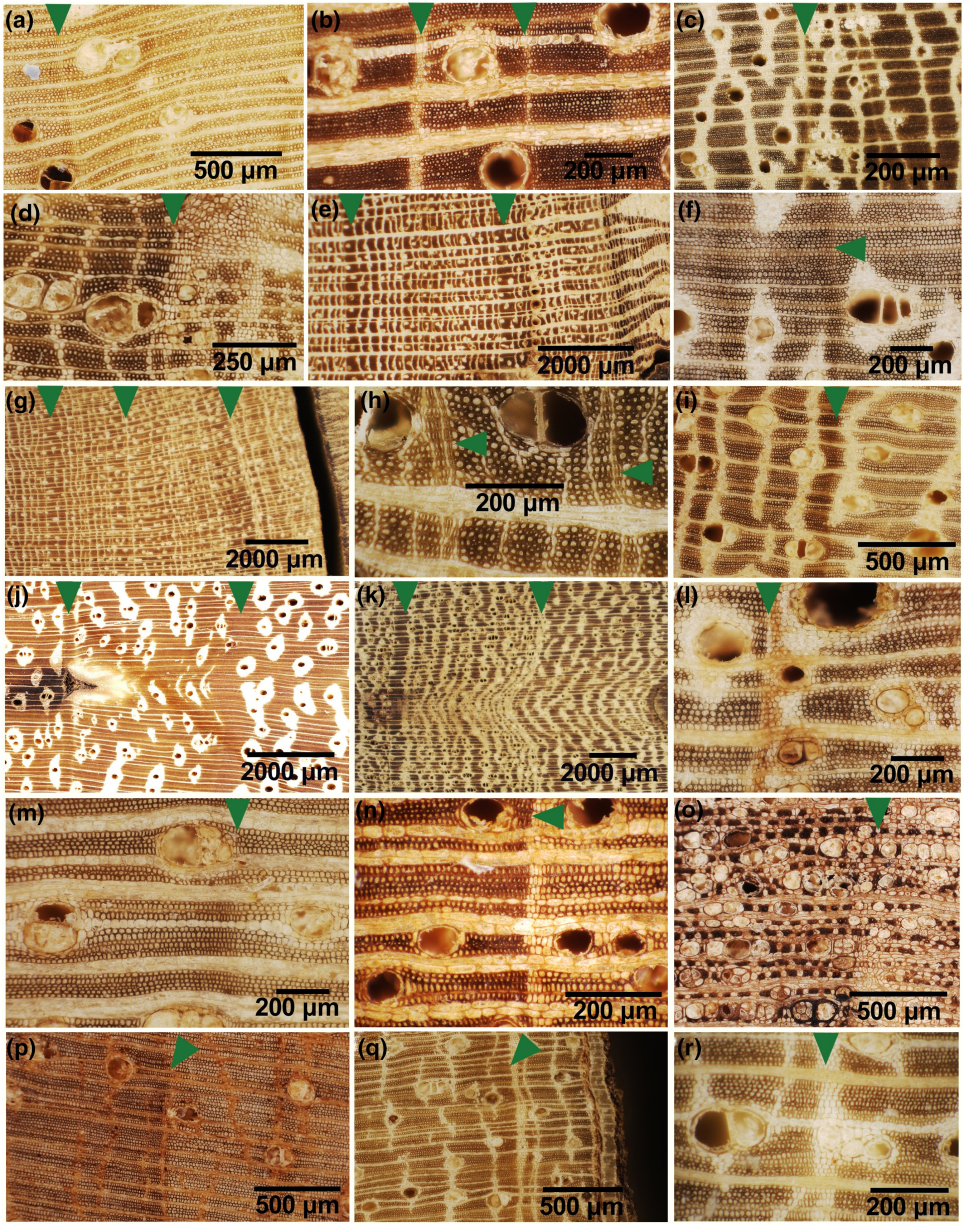
Incident light images of transverse sections showing tree‐ring boundaries (green triangles): (a) *B. welwitschii*, (b) *C. procera*, (c) *C. mildbraedii*, (d) *C. africanum*, (e) *C. griseiflora*, (f) *E. suaveolens*, (g) *G. punctata*, (h) *G. suaveolens*, (i) *L. thompsonii*, (j) *P. macrophylla*, (k) *P. oxyphylla*, (l) *P. macrocarpus*, (m) *P. angolensis*, (n) *S. kamerunensis*, (o) *S. tetrandra*, (p) *T. gilgiana*, (q) *T. prieurieana*, (r) *T. madagascariense*.

All trees in this study, whether deciduous or evergreen, could display growth rings, even though not all growth‐ring boundaries were easily distinguishable, that is, distinct. Only two species (*Carapa procera* (Figure 2b) and *Prioria oxyphylla* (Figure 2k)) always showed distinct rings. The others showed distinct or indistinct rings, varying from one individual to another.

The growth‐ring boundaries we found were marked by one or more of the following structural changes (Table 1): flattened fibers (Figure 2a–d,f,h,i,l,m,n,p,q,r), band (narrow or relatively large) of marginal parenchyma (Figure 2b,d,g,k,l,n,p,q,r), tangential band with little or no apotracheal axial parenchyma (Figure 2e), and tangential band without vessels or axial parenchyma (Figure 2j).

The logistic mixed model to predict growth‐ring boundary distinctness including features DBH, relative mean increment, stratum and leaf phenology class, dormancy periodicity, average dormancy duration, and species nested in site as random effects did not produce a significant model fit. As such, none of the investigated variables aid in the prediction of the distinctness of growth‐ring boundaries.

### 3.2 Interpretation of the number of growth rings observed

The interpretation of the number of growth rings observed and their indication of periodic formation differed between Luki and Yangambi, depending on the sample collection period.

**FIGURE 3:**
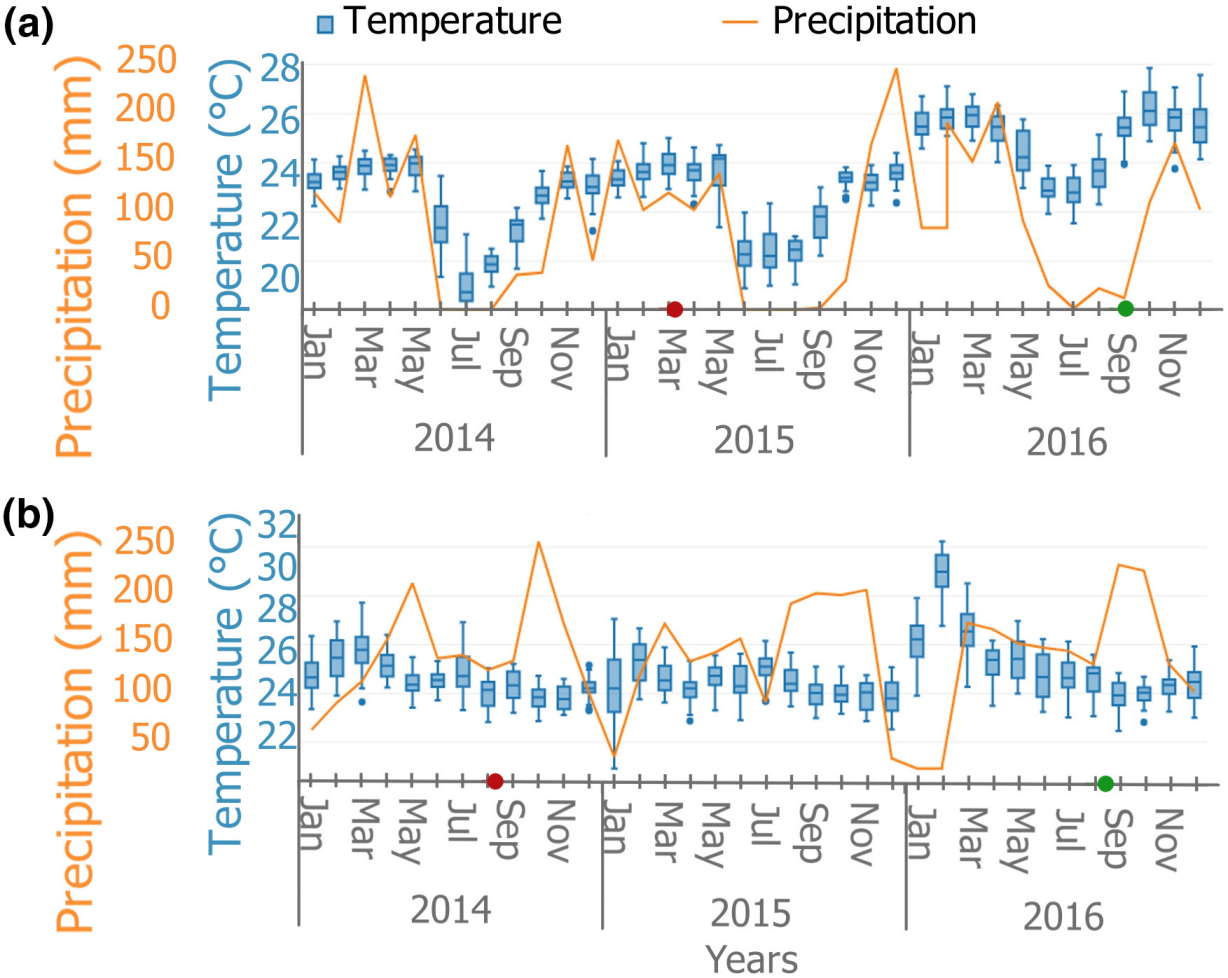
Total monthly rainfall and daily variation of monthly temperature during the study years (2014, 2015, and 2016). (a) Luki and (b) Yangambi. The red and green dots on the timeline mark the marking and collection dates, respectively. Data were obtained from https://power.larc.nasa.gov/data‐access‐viewer/.

The marking in Luki was done within the 2015 wet season (about 2 months before the start of the dry season) and the collection within the 2016 dry season (Figure 3a). As such, the collection was done one and a half seasons after marking. We therefore expect, if growth‐ring formation is annual, to have two ring boundaries formed, the last one at the wood‐bark boundary. In Yangambi, both marking and collection were done during the wet season (August 2014 to August 2016, Figure 3b). Considering that collection was done two seasons after marking, we could expect, if the rings are annual, to have two ring boundaries formed, the last one before the wood‐bark boundary.

**FIGURE 4:**
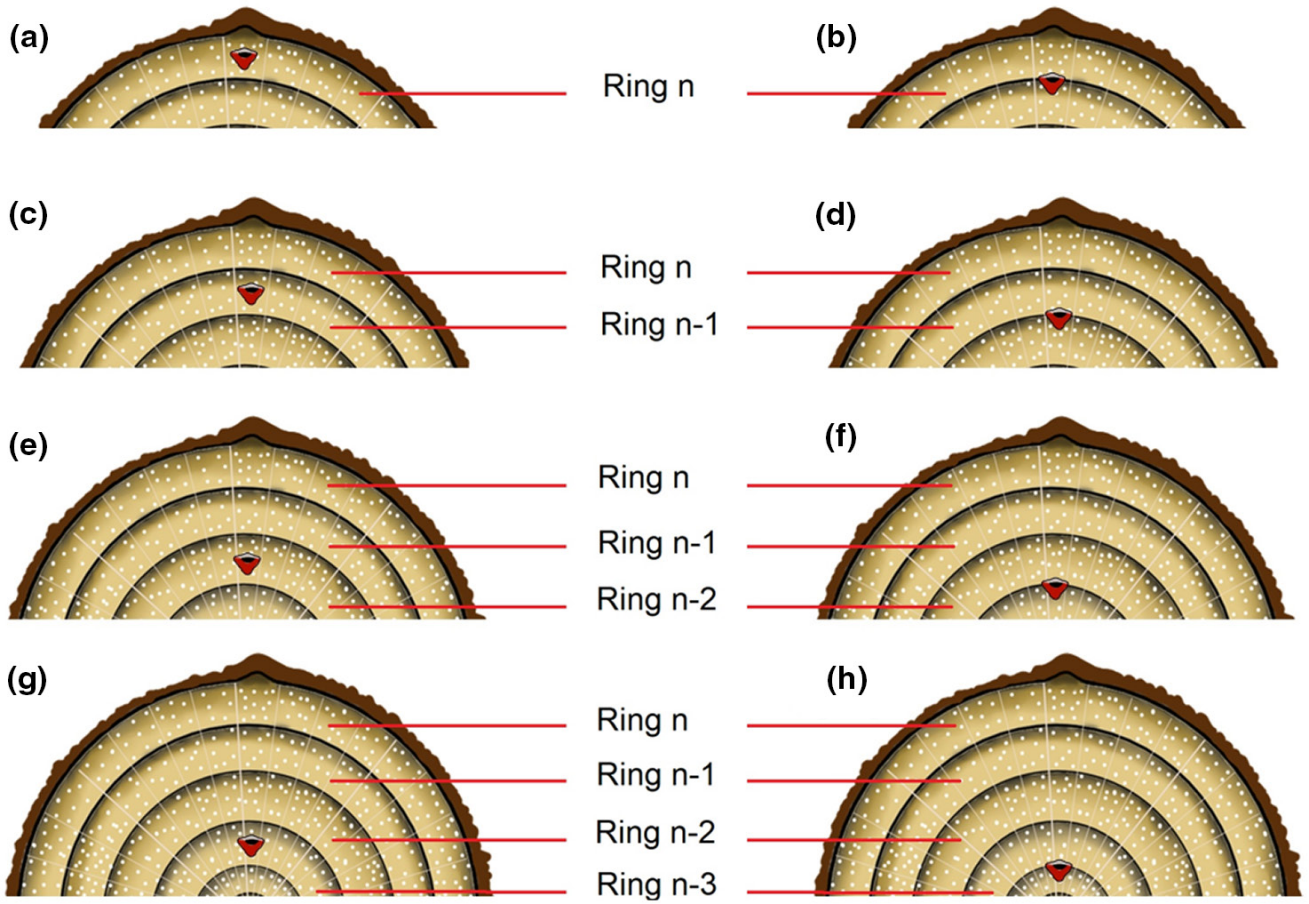
Position of pinning mark on the transverse plane of the stem. (a) Marking inside the last ring, ring *n*; (b) marking at the lower limit of the last ring, ring *n*; (c) marking inside the penultimate ring, ring *n* – 1; (d) marking at the lower limit of the penultimate ring, ring *n* – 1; (e) marking inside the ring preceding the penultimate ring, ring *n* – 2; (f) marking at the lower limit of the ring preceding the penultimate ring, ring *n* – 2; (g) marking inside the *n* – 3 ring; (h) marking at the lower limit of the *n* – 3 ring.

The interpretation of the cambial marking in relation to the growth rings observed during the study period varies between Luki and Yangambi. The following cases can be distinguished: (a) Pinning mark inside the only formed ring, ring *n* (Figure 4a): marking was done while the tree was growing, and collection occurred before or at the beginning of the next growing period began. It is very likely that the single ring formed is not annual. (b) Pinning mark at the lower limit of the last and only formed ring, ring *n* (Figure 4b): Marking was done at the beginning of a growing period, and collection was done before or at the beginning of the next growing period. This case suggests the annual occurrence of ring formation for Luki trees. For Yangambi, there is a high chance that this ring is multiannual. (c) Case like (b) except that the pinning mark is within the penultimate growth ring, the n‐1 ring (Figure 4c). This augurs that the marking was made while the tree was growing. Again, the case highlights the annual occurrence of ring formation for trees in Luki but not for those in Yangambi. (d) Pinning mark at the lower limit of the penultimate ring, ring *n* – 1 (Figure 4d): Marking was done at the beginning of a growing period, and collection was done after the beginning of the next growing period. For Luki, it is possible that the second ring limit was due to lower rainfall in January 2016. For Yangambi, this could suggest that ring formation is annual. (e) Case like (d) except that the pinning mark is inside the ring preceding the penultimate ring, ring *n* – 2 (Figure 4e). This augurs that the marking was made while the tree was growing. Here again, the case shows the occurrence of a single ring per year for Yangambi trees, but two rings for Luki trees. (f) Pinning mark at the lower limit of the ring preceding the penultimate ring, ring *n* – 2 (Figure 4f): marking was done at the beginning of a growing period, and collection was done after the beginning of two supplementary growing periods. This case does not exist for the Luki samples; in Yangambi, it suggests the possibility of several growth rings in some years. (g) Case like (f) except that the pinning mark is located inside the *n* – 3 ring (Figure 4g). This augurs that the marking was made while the tree was growing. (h) Pinning mark located at the lower limit of the *n* – 3 ring (Figure 4h). Marking was done at the beginning of a growing period, and collection was done after the beginning of tree supplementary growing periods. This case does not exist in the Luki samples either. For the Yangambi samples, it suggests the possibility of occurrence of two growth rings per year.

**FIGURE 5:**
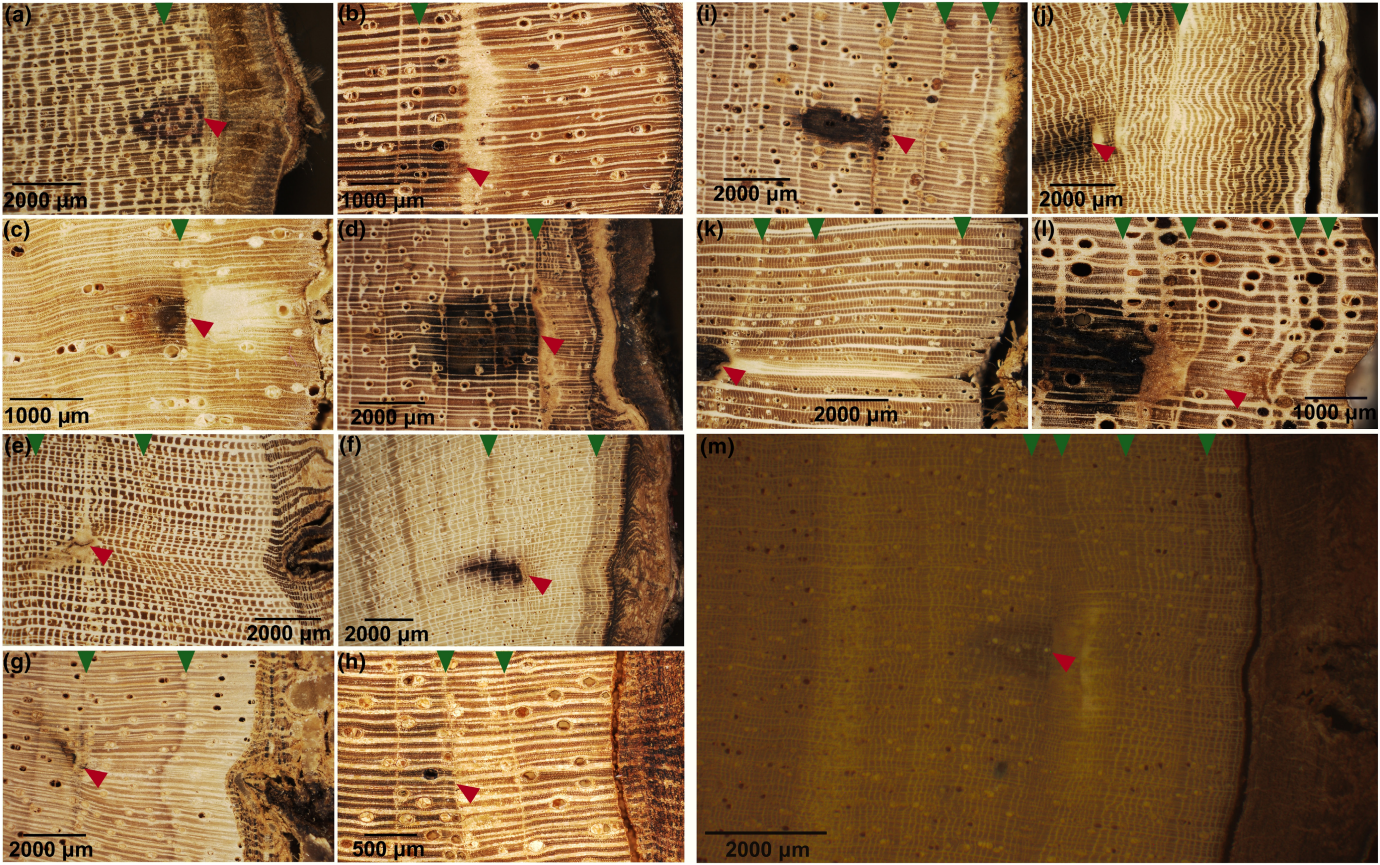
Images of wood samples showing the limits of growth rings (green triangles) and the new wood layers containing the scar (red triangle) resulting from cambial marking: (a) *P. macrocarpus*, (b) *C. procera*, (c) *B*. *welwitschii*, (d) *P. oxyphylla*, (e) *C. griseiflora*, (f) *T. madagascariense*, (g) *P. angolensis*, (h) *S. kamerunensis*, (i) *P. oxyphylla*, (j) *C. mildbraedii*, (k) *G. suaveolens*, (l) *P. oxyphylla*, and (m) *C. africanum*.

These cases of cambial markings in relation to observed growth‐ring boundaries were found in the sampled trees at Luki and Yangambi, with a few species‐specific examples presented here (Figure 5). Case (a) showing the pinning mark inside the last formed growth ring was found, as in species *P. macrocarpus*, from Luki (Figure 5a) and in *C. procera* from Yangambi (Figure 5b). The case (b) showing the pinning mark at the lower limit of the last ring, ring n, was found, for example, in *B. welwitschii* (Figure 5c) from Luki, and in *P. oxyphylla* (Figure 5d) from Yangambi. The case C, showing the pinning mark inside the penultimate ring, ring *n* – 1, was found, for example, in *C. griseiflora* from Luki (Figure 5e), and in *T. madagascariense* from Yangambi (Figure 5f). The case d, showing the pinning mark at the lower limit of the penultimate ring, ring *n* – 1, was found, for example, in *P. angolensis* from Luki (Figure 5g) and in *S. kamerunensis* from Yangambi (Figure 5h). The case E, showing the pinning mark inside the ring preceding the penultimate ring, ring *n* – 2, was found, for example, in *P. oxyphylla* from Luki (Figure 5i) and in *C. mildbraedii* from Yangambi (Figure 5j). The case F, showing the pinning mark at the lower limit of the ring preceding the penultimate ring, ring *n* – 2, was found, for example, in *G. suaveolens* from Yangambi (Figure 5k). The case G, showing the pinning mark inside the *n* – 3 ring, was found, for example, in *P. oxyphylla* from Yangambi (Figure 5l). The case H, showing the pinning mark at the lower limit of the n‐3 ring, was found, for example, in *C. africanum* from Yangambi (Figure 5m).

### 3.3 Periodicity of growth‐ring formation

Nine species in Luki (*B. welwitschii*, *C. griseiflora*, *C. procera*, *G. suaveolens*, *P. angolensis*, *P. oxyphylla*, *S. kamerunensis*, *S. tetrandra*, *T. gilgiana*) and two species in Yangambi (*P. angolensis and T. gilgiana*) formed growth rings periodically in all their individuals. In Yangambi, four species (*C. griseiflora*, *C. procera*, *P. macrophylla,* and *P. oxyphylla*) did not form periodical growth rings in any of the studied individuals. In the remaining species, the periodical formation of growth rings varied from one individual to another. This intra‐species variability was observed in 9 species in Luki and 12 species in Yangambi.

The logistic mixed model to predict growth‐ring periodicity including parameters DBH, relative mean increment and site as fixed effects and species nested in site as random effects produced the best fit:

$$\begin{array}{c} \text{periodicity}\;\left(\text{binary}\right)\sim \mathrm{DBH}+\text{Relative mean increment}\\ \;+\text{Site}+\left(1|\text{Site}/\text{Species}\right). \end{array}$$

The model's explanatory power related to the fixed effects alone (marginal R2) is 0.72. A significant positive effect of DBH (beta = 0.03, 95% CI [6.43e‐04, 0.07], *p* = .046; SD beta = 0.56, 95% CI [0.01, 1.11]) and relative mean increment (beta = 1.91, 95% CI [1.12, 2.69], *p* < .001; SD Beta = 2.43, 95% CI [1.43, 3.44]) was found, while a significant negative effect of site [Yangambi] was observed (beta = −3.52, 95% CI [−5.24, −1.79], *p* < .001; SD beta = −3.52, 95% CI [−5.24, −1.79]) (Table 2).

**TABLE 2:** Model output of quantifying growth‐ring periodicity in response to DBH (initial, at start of observation period), relative mean increment, and site as fixed effects, using a generalized linear mixed‐effects model.

Predictors	Estimate	SE	*z*	*p*	Significant
Intercept	−1.01079	0.72134	−1.401	.1611	
DBH (initial)	0.0339	0.01697	1.998	.0457	*
Relative mean increment	1.90599	0.40122	4.75	<.001	***
Site: Yangambi	−3.51591	0.88037	−3.994	<0.001	***

Random effect	Variance	SD			
Species within site	2.05	1.432			

*Note*: Tree species are nested within site as a random effect (Significant codes: 0 ‘***’ 0.001 ‘**’ 0.01 ‘*’ 0.05 ‘.’ 0.1 ‘’ 1).

The species‐level classifications of stratum, phenology, or the phenological parameters (phenophase duration or periodicity) did not significantly contribute to the prediction of growth‐ring periodicity or improved the model fit.

## 4 DISCUSSION

### 4.1 Distinctness of growth ring

The results of this study indicate that all eighteen species examined can form growth rings, demarcated by the following features, some observed simultaneously within a same species: flattened fibers, one or two layers of marginal parenchyma, tangential band with little or no apotracheal axial parenchyma, or tangential band without vessels or axial parenchyma. These anatomical features are among those used by several authors to formally identify the ring boundary in some tropical trees (Détienne, 1989; Détienne et al., 1998; Mariaux, 2016; Robert et al., 2011; Worbes, 1999). We found that not all growth‐ring boundaries were distinct. Ring boundaries of 43.3% of trees showing rings were indistinct. However, the logistic mixed model we fitted revealed that no variables including DBH, mean relative increment, leaf stratum and phenological class, dormancy periodicity, mean dormancy duration, species, and site, predicted the distinctness of growth‐ring boundaries.

Growth rings are supposed to form when the tree is dormant as a result of an environmental stress factor (Worbes, 2002). The absence or limited seasonality in climatic variables in the tropics has been the basis for the long‐held belief that tropical trees exhibit relatively constant growth throughout the year and do not form growth rings (Brienen et al., 2016; Lieberman & Lieberman, 1985). It is well known today that many tropical trees form distinct growth rings and that rainfall seasonality (Gourlay, 1995; Worbes, 1995, 1999), temperature (Singh & Kushwaha, 2016), and light (Tarelkin et al., 2019) are by far the most common limiting factors for tropical tree growth. Complex interactions between the tree's organic functions, including transpiration, water uptake and storage, and environmental variables, including precipitation, sunlight, temperature, and soil water reserve, lead to phenological response patterns specific to each species, and even to each tree (Borchert, 1999; Singh & Kushwaha, 2016). Light intensity has been shown to have the greatest influence on the formation of growth‐ring boundaries during the dry season. The reduction in daylight length, albeit slight in the tropics, and thick cloud cover during the dry season adversely affect the amount of sunlight in terms of duration and intensity (Tarelkin et al., 2019; Vargas et al., 2022). This can lead to an intensification of leaf fall, which in turn also negatively affects the wall thickness and size of the cells formed in the cambium (Janssen et al., 2021).

Growth‐ring distinctness was however not significantly related to any of the investigated variables, including DBH, relative mean increment, tree stratum, leaf phenology, mean leaf phenophase duration, leaf phenophase periodicity, and site. Previous studies do however show a relation between growth‐ring distinctness and leaf phenological classes (Worbes, 1999). Indeed, Worbes (1999) found, in a study conducted in Venezuela, that ring boundaries, when present, are more pronounced in deciduous than in evergreen species. He postulated that evergreen species tend to show only a short interruption in wood growth during the latter part of the dry season, whereas deciduous species stop growing completely at the end of the rainy season.

In our case, we believe there was a bias due to differences in microclimate that were not considered. Intra‐species variability in leaf phenology is high (Kearsley et al., 2024) and will depend on both internal cues and environmental drivers. The phenological observations available for this study did not account for the growing conditions of the individual trees (e.g., location in the canopy, gap effects, and neighboring effects) or for the weather during the observation period (how severe was the dry season, what were the light conditions). When repeating this experiment, we recommend the collection of simultaneous phenological observations.

### 4.2 Periodicity of growth‐ring formation

We found that site was one of the main predictors of growth‐ring periodicity. Only nine species from Luki (B*. welwitschii*, *C. griseiflora*, *C. procera*, *G. suaveolens*, *P. angolensis*, *P. oxyphylla*, *S. kamerunensis*, *S. tetrandra*, and *T. gilgiana*) and two species from Yangambi (*P. angolensis* and *T. gilgiana*) had all individuals forming growth rings periodically. Five species, namely, *C. griseiflora*, *C. procera*, *G. suaveolens*, *P. oxyphylla,* and *S. tetrandra*, behaved differently depending on the site: They show exclusively periodic behavior in Luki, but irregular behavior in Yangambi. Two species, namely, *P. angolensis* and *T. gilgiana,* displayed an exclusively periodic behavior in both sites.

The disposition to form growth rings is due to the fluctuation of environmental variables such as precipitation, temperature, soil moisture, air humidity, and sunlight. The more marked and seasonal the variations, the more periodic the growth rings (Brienen et al., 2016; Janssen et al., 2021). In contrast to temperate and boreal regions, environmental variables in the tropics are less distinctly seasonal. As a result, temporal morphological markers are either absent, irregular, or unclear (Brienen et al., 2016), depending on the severity and length of arid periods (Worbes, 2002). Accordingly, in Yangambi where the dry season is less pronounced, trees have a lower likelihood for periodical growth‐ring formation than trees in Luki. Similar observations were reported in several previous studies (Coster, 1927; Pearson et al., 2011). Nevertheless, even in Luki where a distinct dry season is present, the formation of growth rings is not always periodic. Indeed, Luki's hilly landscape and proximity to the Atlantic Ocean cause frequent fog and dense cloud cover that keeps relative humidity high throughout the year (Lubini, 1997; Sénéchal et al., 1989). Therefore, despite relatively low annual rainfall during the three to four months of the dry season (less than 60 mm of rainfall per month), plants do not experience extreme water stress during this period (Couralet et al., 2013; Lubini, 1997).

The nine remaining species, namely, *C. africanum*, *C. mildbraedii*, *E. suaveolens*, *G. punctata*, *G. thompsonii*, *P. macrocarpus*, *P. macrophylla*, *T. madagascariense,* and *T. prieurieana*, had an irregular behavior in both sites: some trees formed growth rings periodically and others irregularly, in the same site. This may depend on the microclimate of each individual due to its location in the forest (stratum, soil quality, altitude, etc.), and its own sensitivity. Mariaux (2016) reported that within a species, ring formation can be dependent on the personal life history of individuals (forest fire, damage to the crown, insect attacks, etc.). The periodicity of ring formation may also differ between life stages. Clear, annual rings may be found in the adult stages, but absent, vague, or non‐annual rings in the juvenile stages (Brienen & Zuidema, 2005; Dünisch et al., 2002).

We found that relative mean increment was also a main predictor of growth‐ring periodicity. Trees with higher relative mean increment have a higher likelihood of having periodical growth rings. In other words, fast‐growing trees have a higher chance of periodical growth rings. Previous studies supported that in medium and fast‐growing trees, it is usually easy to see the annual growth rings (Détienne, 1989; Ogden & West, 1981; Verheyden et al., 2004). Fast‐growing trees are often light‐demanding (Marra et al., 2014). As such, their growth is more dependent on high light availability. As in the dry season, day length is reduced and there is a cloud cover that reduces the intensity of sunlight in the study sites (Tarelkin et al., 2019), and fast‐growing species are more prone to a reduction or outright cessation of growth that induces the formation of a sharp, periodic ring boundaries. Things are different for slow‐growing understory species. It has been shown that in western Central Africa, home to light‐poor evergreen forests, significant low‐level cloud cover during the dry season from June to September results in a sharp reduction in water demand and an improvement in the quality of light available for tree photosynthesis (Philippon et al., 2019). This improvement in light conditions is of particular benefit to evergreen understory species, which find opportunities to access light when the canopy opens due to the fall of deciduous tree leaves during the dry season (Denslow, 1987).

We found that DBH had a significant positive effect, although a small estimate, on the growth‐ring periodicity. Since DBH is related to the height of a tree, DBH can be used as a proxy for a trees' location within the canopy, and as such for light, it can receive. So, the signal is potentially larger for trees higher up in the canopy (López et al., 2012). The life history stratum classification of a tree species was, however, not a predictor for growth‐ring periodicity. Stratum classification at species‐level does not represent the growing conditions of an individual tree.

We expected a clear link with phenology; however, the phenological classification and parameters used were not specifically observed for the individual trees under investigation. And indeed, no link was established. Nevertheless, deciduous species, which are more sensitive to water stress than evergreen species, could be more inclined to form growth‐ring boundaries not only during the dry season, but also during all periods characterized by a considerable drop in rainfall (such as the period from January to February in Luki and June–July in Yangambi). Previous studies already reported strong differences in species sensitivity to precipitation, due to water storage in the stems, phenology, rooting depth, and reserve utilization (Borchert, 1994; Meinzer et al., 1999). In situ observations of leaf phenology of the studied trees might provide more insight. At the site level, the fact that not all trees of deciduous species form the same number of growth rings even though they grow on the same site confirms the individual specificity in the formation of growth rings due to local growing conditions (canopy/understory, gap/no‐gap, neighboring trees, etc.), as supported by Mariaux (2016).

## 5 CONCLUSION

In the present study, we examined ring formation in eighteen tree species growing under two different climatic conditions in the semi‐deciduous rainforests of the Luki and Yangambi biosphere reserves in the Congo Basin. The study showed that all eighteen species formed growth rings. The periodicity of ring formation varied significantly within and between species, as well as between sites, while ring boundary distinctness was not significantly related to any parameter at either site. The formation of annual growth rings is more likely at Luki than at Yangambi, where climate seasonality is less marked. Inter‐species variability in the formation of periodic growth rings is not related to species‐level classifications of leaf phenology. Environmental conditions at site and tree level, as well as microclimate, may dictate intra‐ and inter‐species variability. These results clearly demonstrate that cambial marking can be successfully applied in the Congo Basin. Since the absence of wood anatomically distinct borders between growth rings, a high‐resolution assessment of the secondary growth needs to be done with periodic sampling of the cambial zone through microcores. This approach would allow to date the formation of tissue types and production of wood and add to phenological datasets.

## CONFLICT OF INTEREST STATEMENT

The authors declare that the research was conducted in the absence of any conflict of interest.

## Supporting information


**Data S1:** Supporting information.

## Data Availability

The data that support the findings of this study are openly available (Belanganayi, 2024) in figshare at https://doi.org/10.6084/m9.figshare.25167212 and https://doi.org/10.6084/m9.figshare.25507546.
