# Supplementary material for: Growth periodicity in semi‐deciduous tropical tree species from the Congo Basin
Source: Plant Environ Interact. 2024 May 22;5(3):e10144. doi: 10.1002/pei3.10144 (PMC11112140; doi:10.1002/pei3.10144)
Supplement: Supplementary file 1 — Data S1: Supporting information. [file PEI3-5-e10144-s001.docx]

**Supporting Information**

Article title: **Growth periodicity in semi-deciduous tropical tree species from the Congo Basin**

**Tropical Tree Growth: Congo Basin**

Basile Luse Belanganayi, Claire Delvaux, Elizabeth Kearsley, Kévin Lievens, Mélissa Rousseau, Christophe Mbungu Phaka, Brice Yannick Djiofack, Félix Laurent, Nils Bourland, Wannes Hubau, Tom De Mil, Hans Beeckman

The following Supporting Information is available for this article:

**Fig. S 1:** Wood samples marked, sanded, and mounted on a numbered support from the Tervuren wood (Tw) xylotheque.

**Fig. S 2:** Capturing an image of a marked wood sample using an HRX-01 3D Digital Microscope.

**Tab. S 1:** Species-by-species comparison of wood samples from Luki and Yangambi.


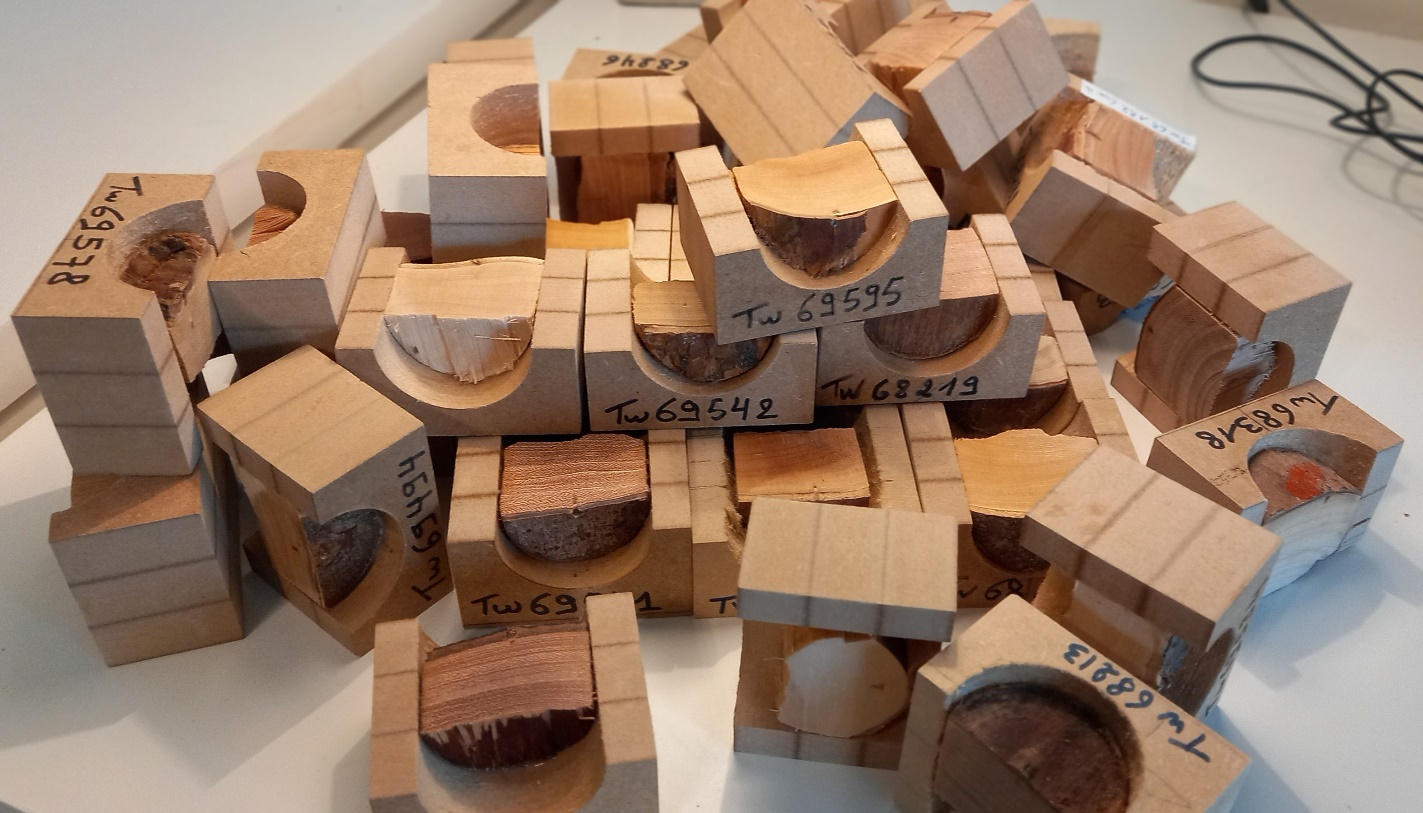


Figure S 1: Wood samples marked, sanded, and mounted on a numbered support from the Tervuren wood (Tw) xylotheque.


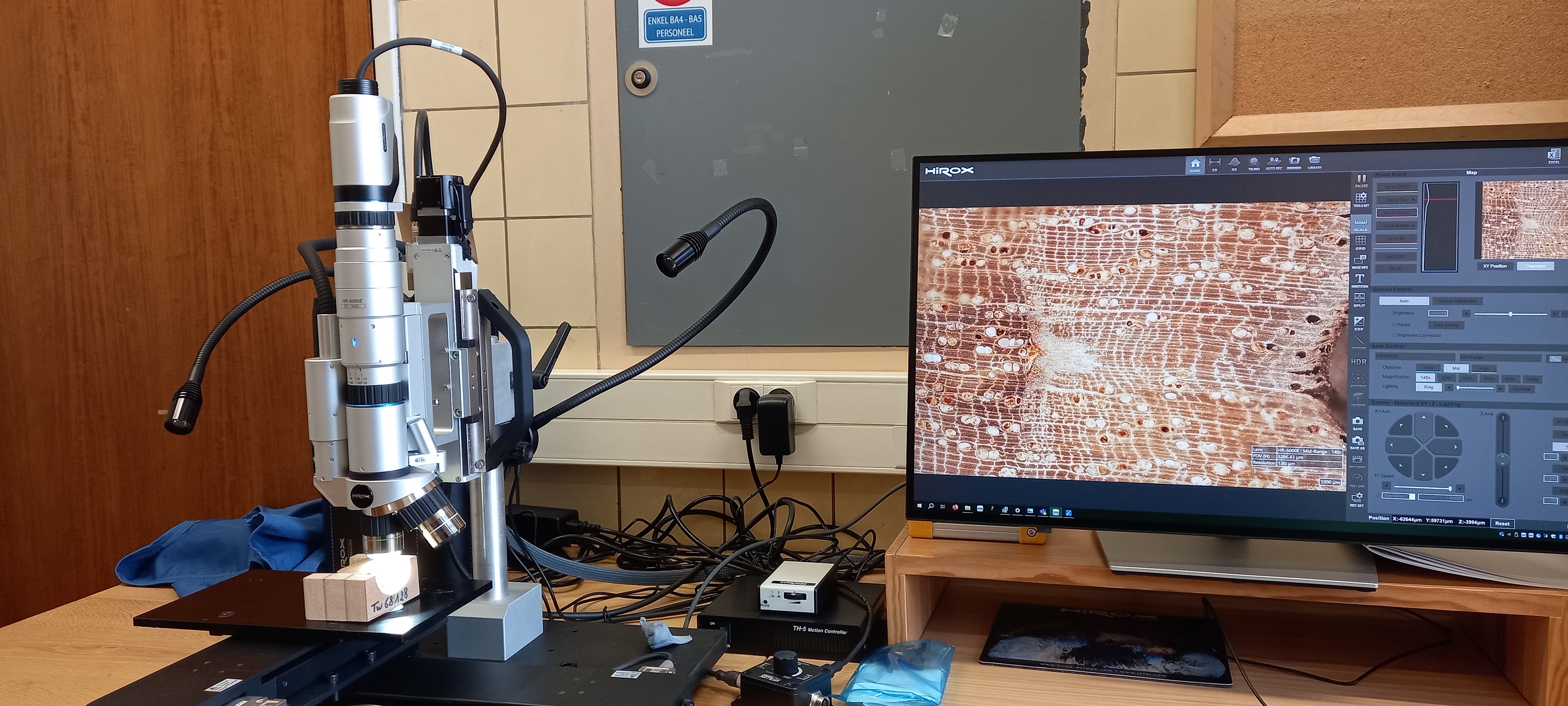


Figure S 2: Capturing an image of a marked wood sample using an HRX-01 3D Digital Microscope.

Table S 1: Species-by-species comparison of wood samples from Luki and Yangambi.

*Blighia welwitschii*

| 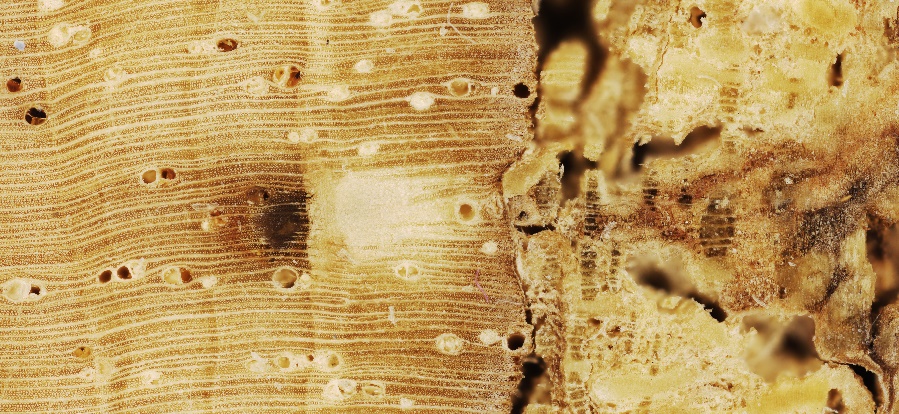  Tw68081 | 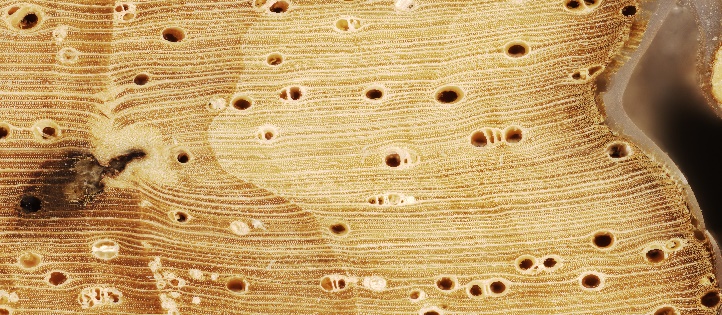  Tw69568 |
| --- | --- |

*Carapa procera*

| 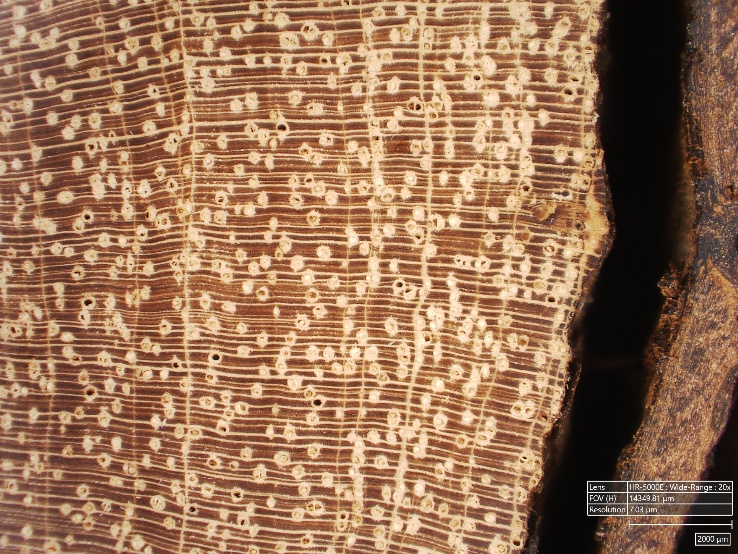  Tw68091 | 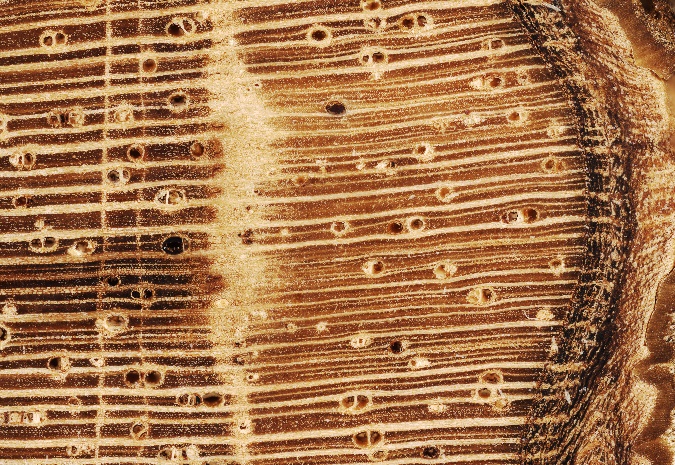  Tw69551 |
| --- | --- |

*Celtis mildbraedii*

| 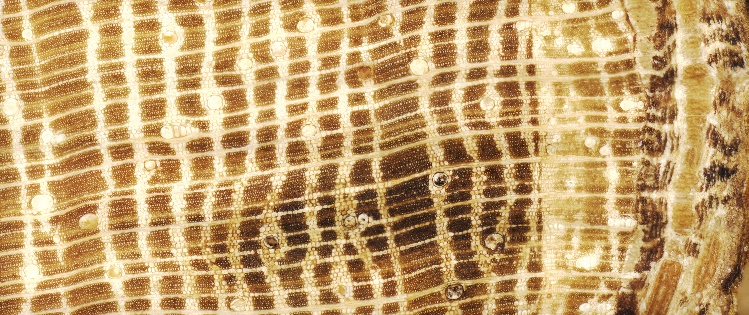  Tw68103 | 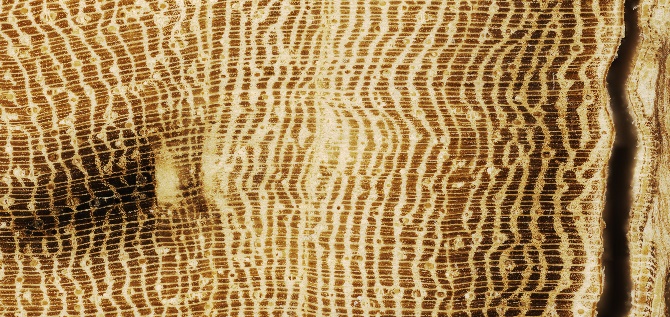  Tw69494 |
| --- | --- |

*Chrysophyllum africanum*

| 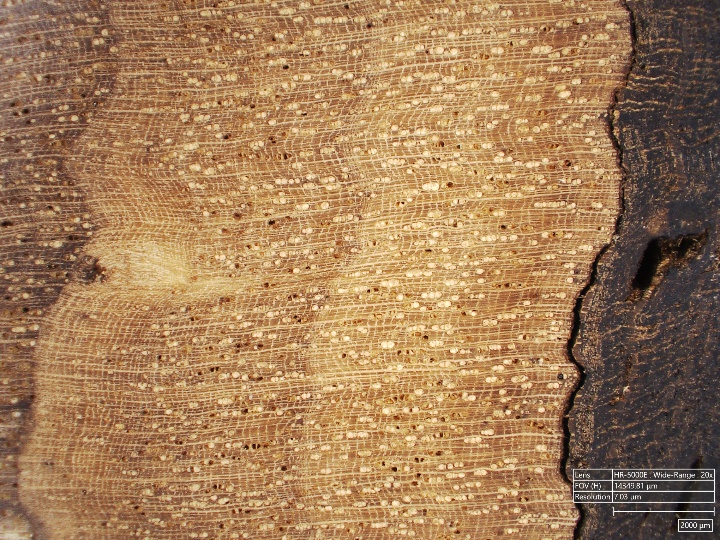  Tw68120 | 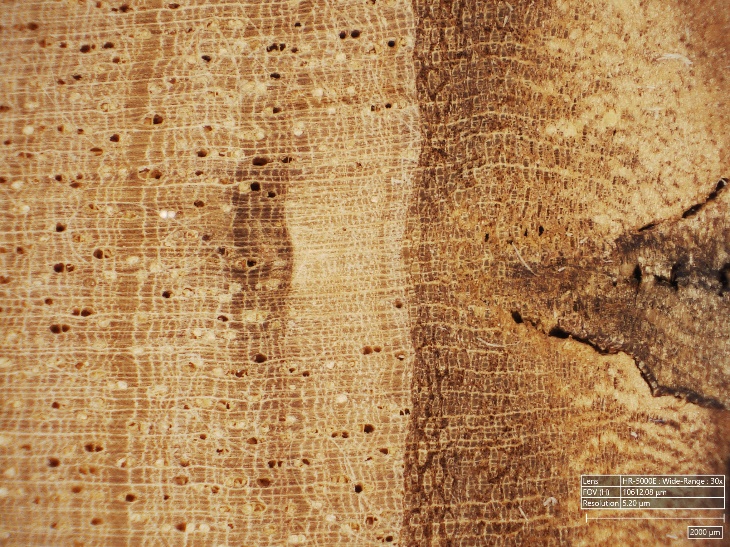  Tw69517 |
| --- | --- |

*Cola griseiflora*

| 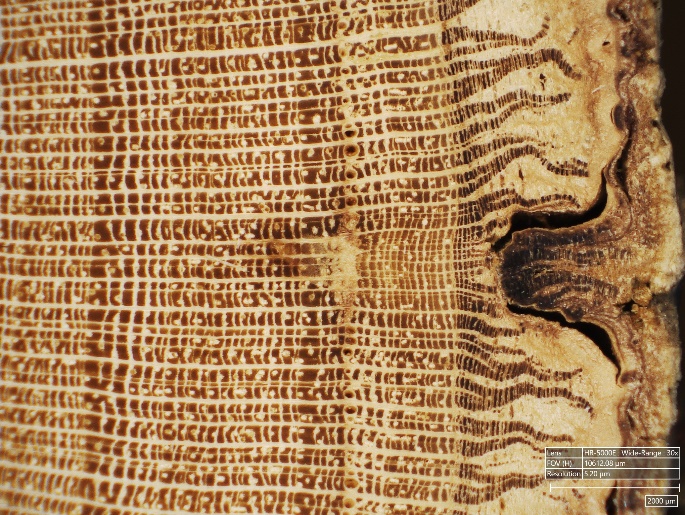  Tw68134 | 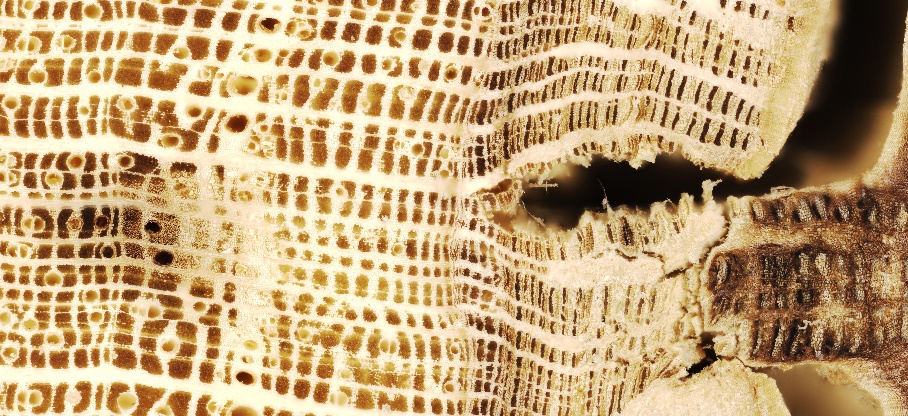  Tw69597 |
| --- | --- |

*Erythrophleum suaveolens*

| 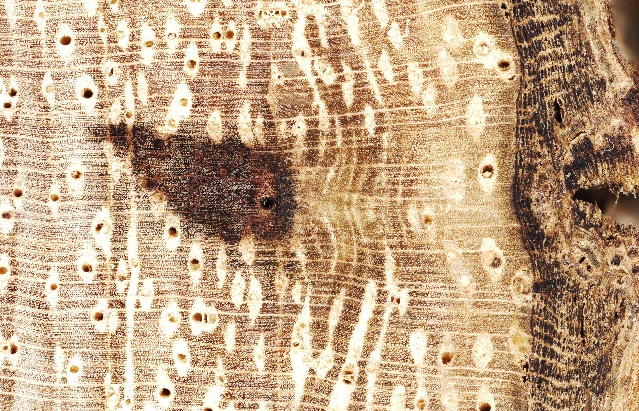  Tw68145 | 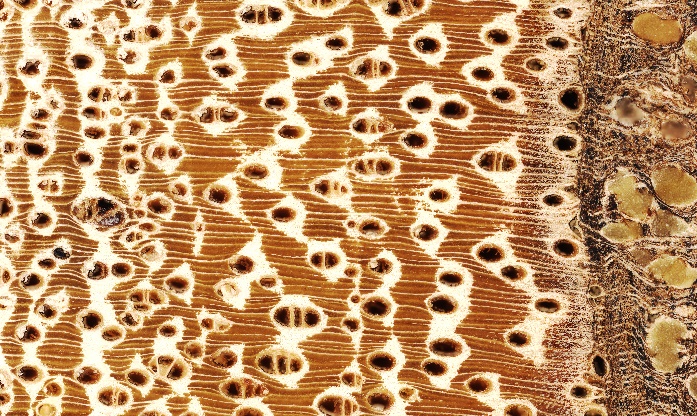  Tw69544 |
| --- | --- |

*Garcinia punctata*

| 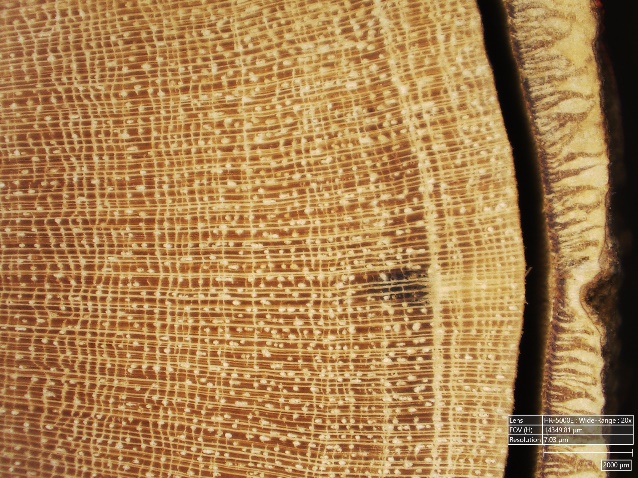  Tw68155 | 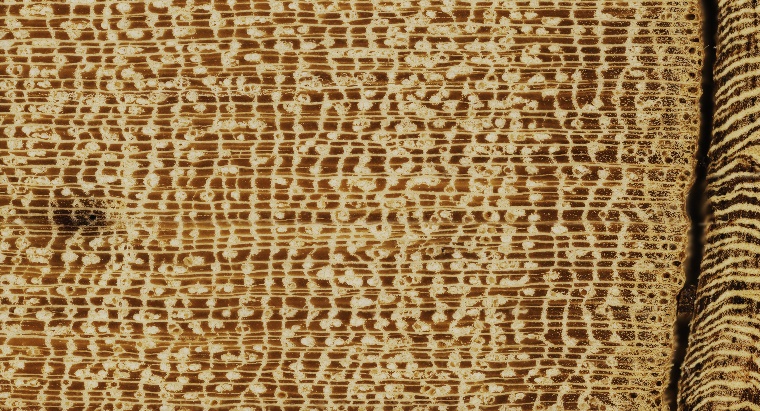  Tw69542 |
| --- | --- |

*Greenwayodendron suaveolens*

| 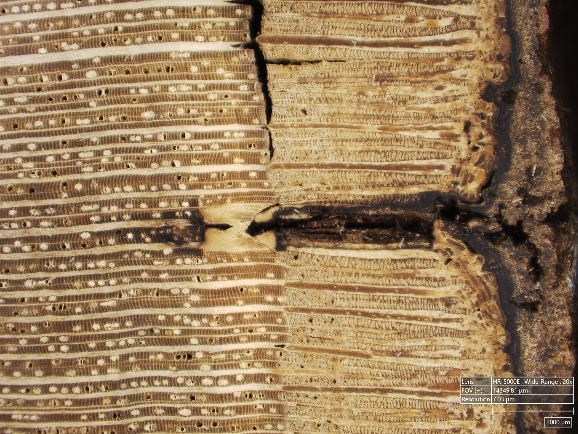  Tw68175 | 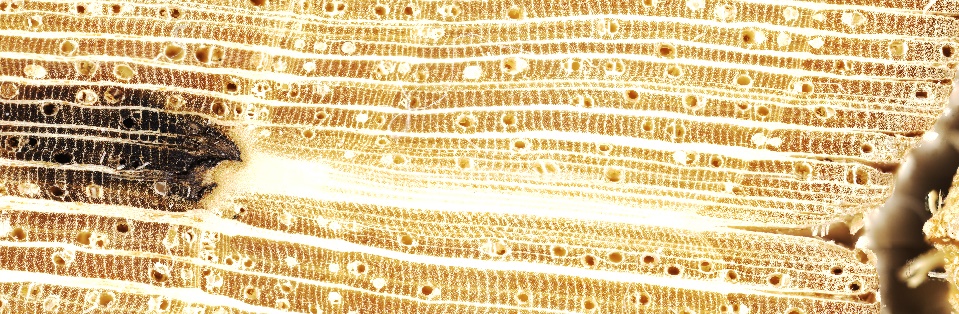  Tw69480 |
| --- | --- |

*Leplaea thompsonii*

| 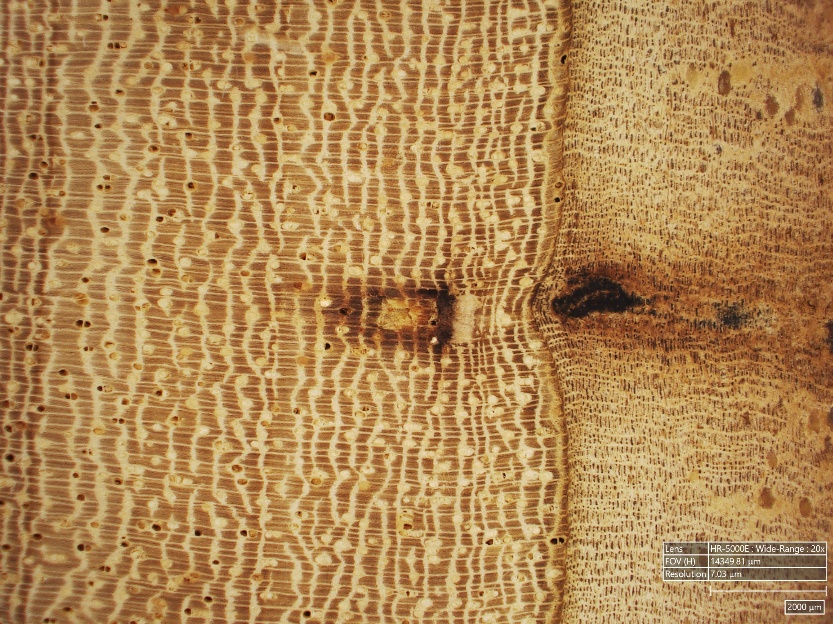  Tw68184 | 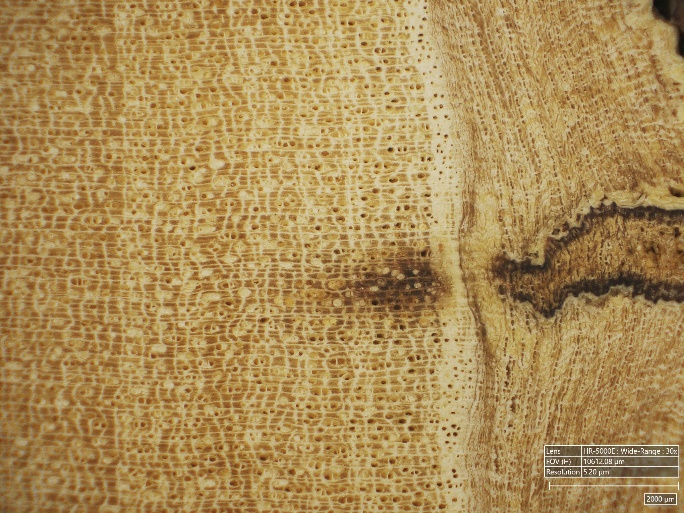  Tw69534 |
| --- | --- |

*Pentaclethra macrophylla*

| 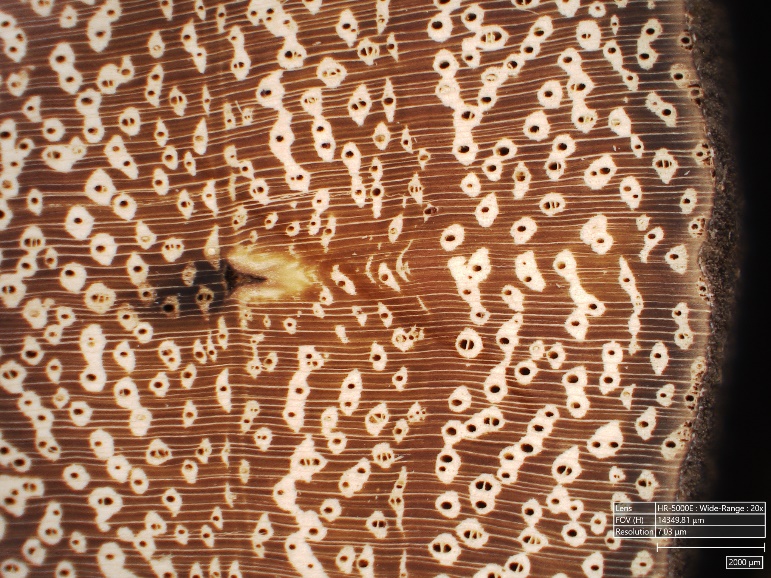  Tw68189 | 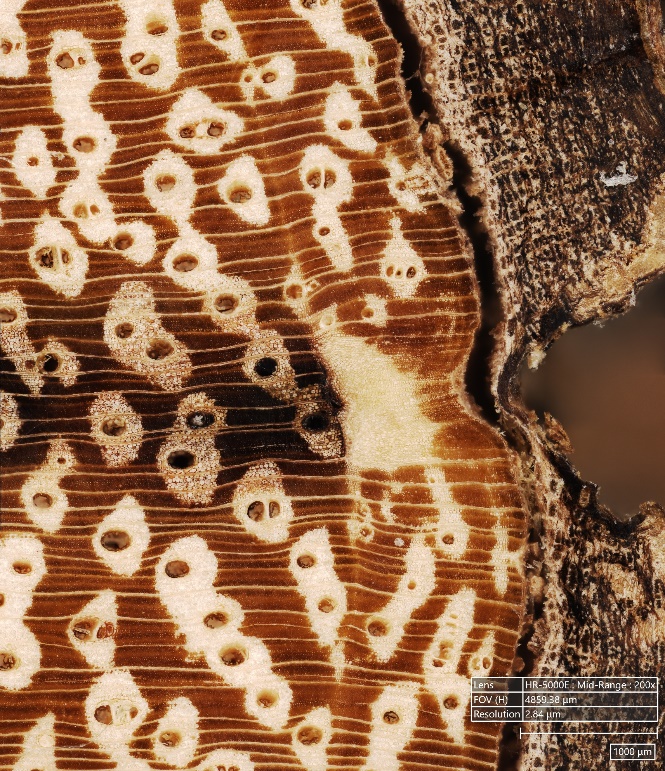  Tw69541 |
| --- | --- |

*Petersianthus macrocarpus*

| 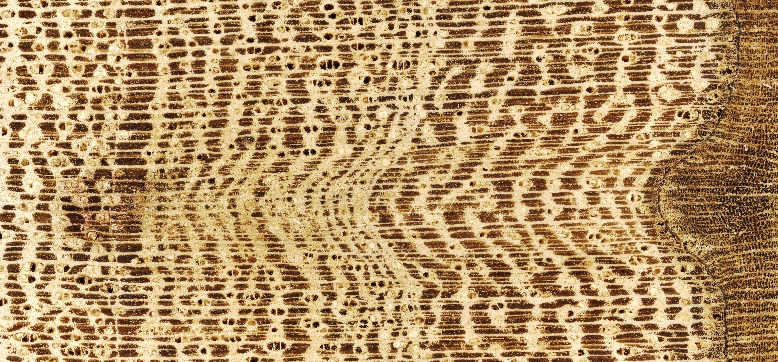  Tw68204 | 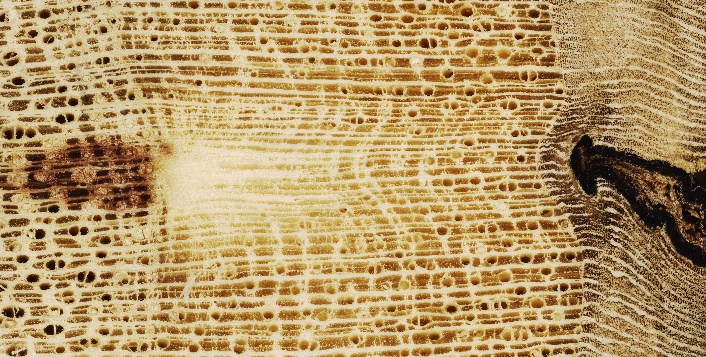  Tw69578 |
| --- | --- |

*Prioria oxyphylla*

| 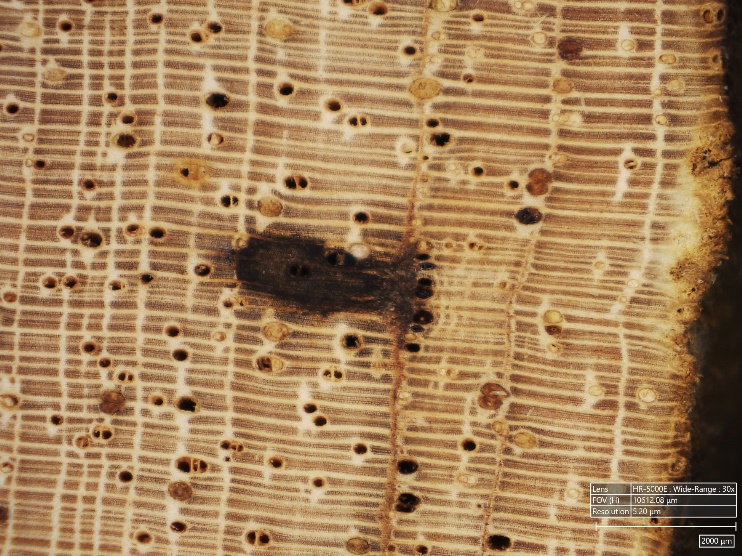  Tw68219 | 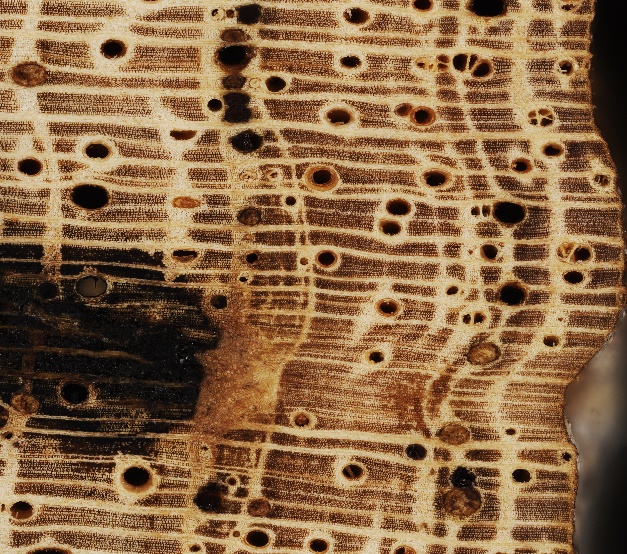  Tw6925 |
| --- | --- |

*Pycnanthus angolensis*

| 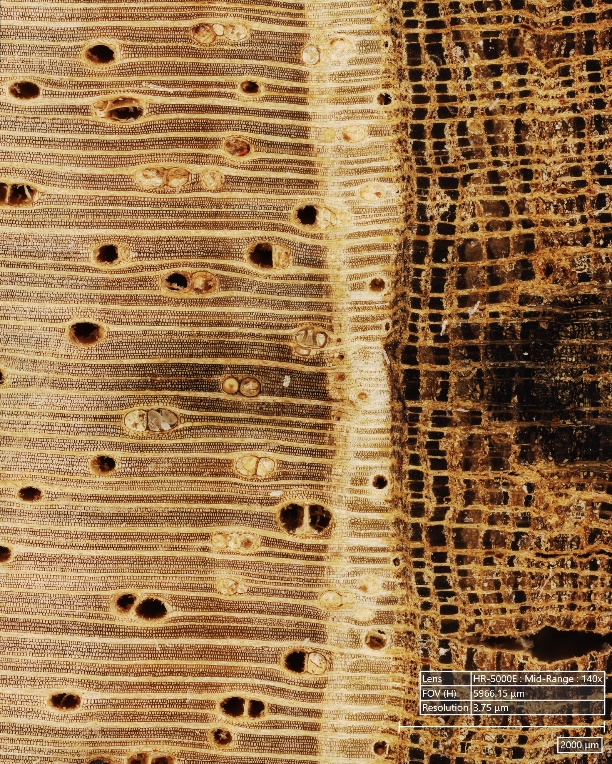  Tw68230 | 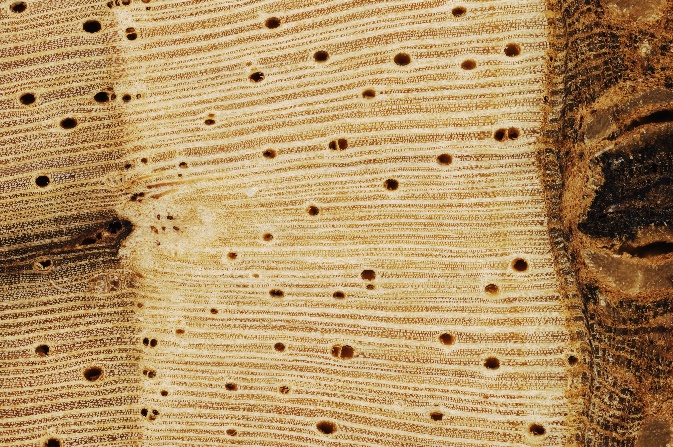  Tw69565 |
| --- | --- |

*Staudtia kamerunensis var gabonensis*

| 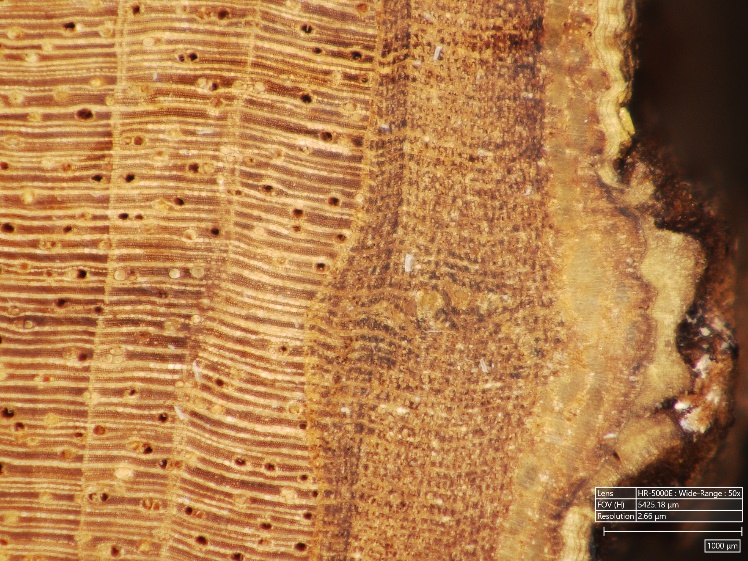  Tw68244 | 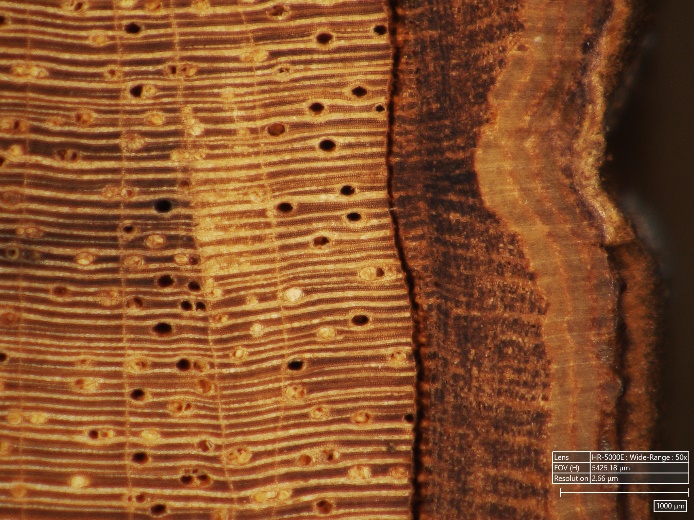  Tw69511 |
| --- | --- |

*Strombosiopsis tetrandra*

| 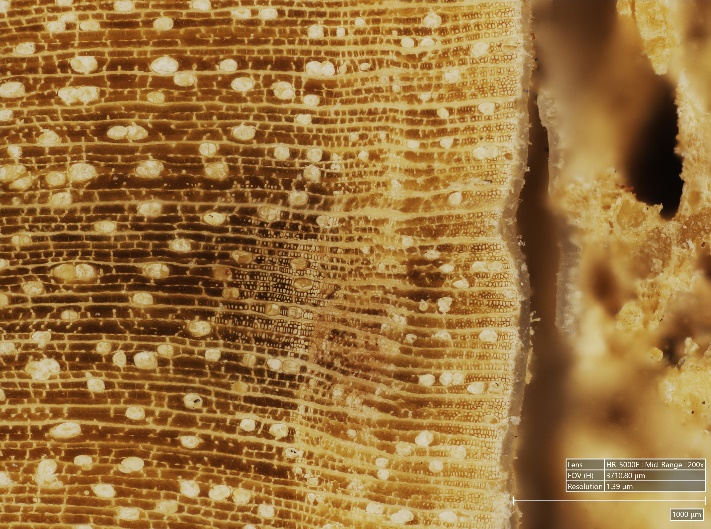  Tw68249 | 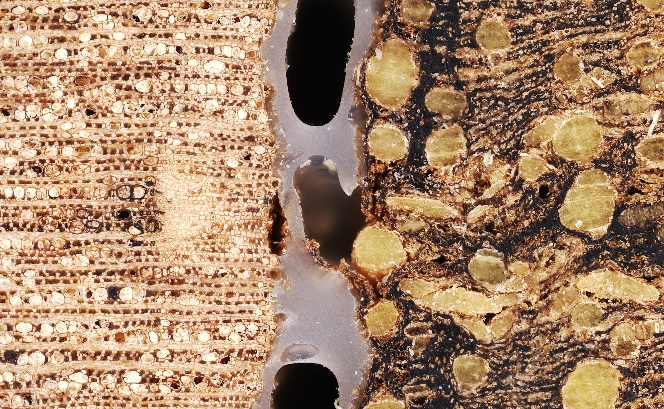  Tw69490 |
| --- | --- |

*Trichilia gilgiana*

| 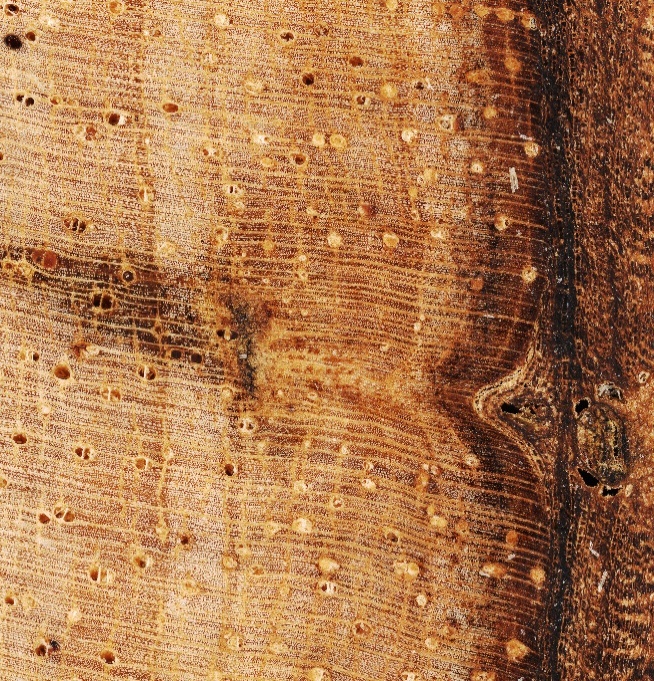  Tw68259 | 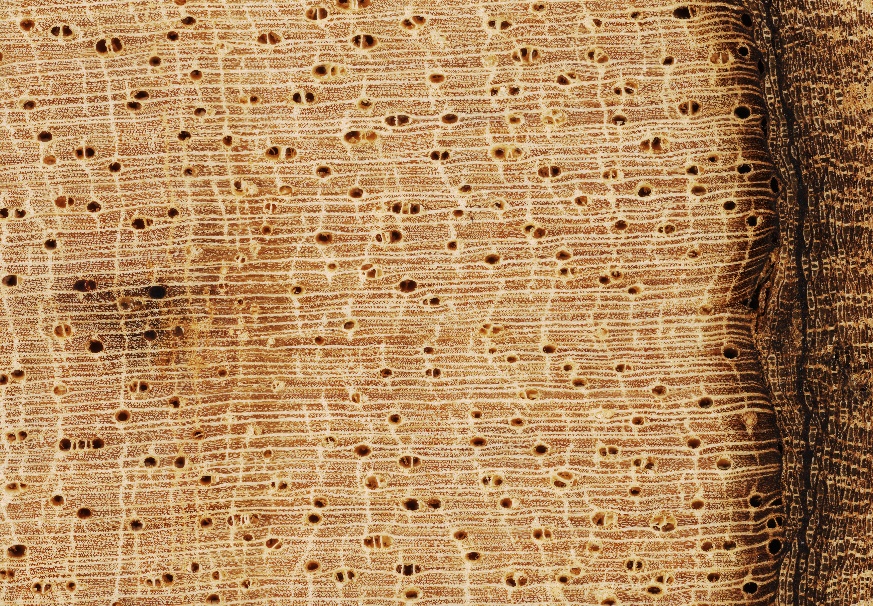  Tw69594 |
| --- | --- |

*Trichilia prieurieana*

| 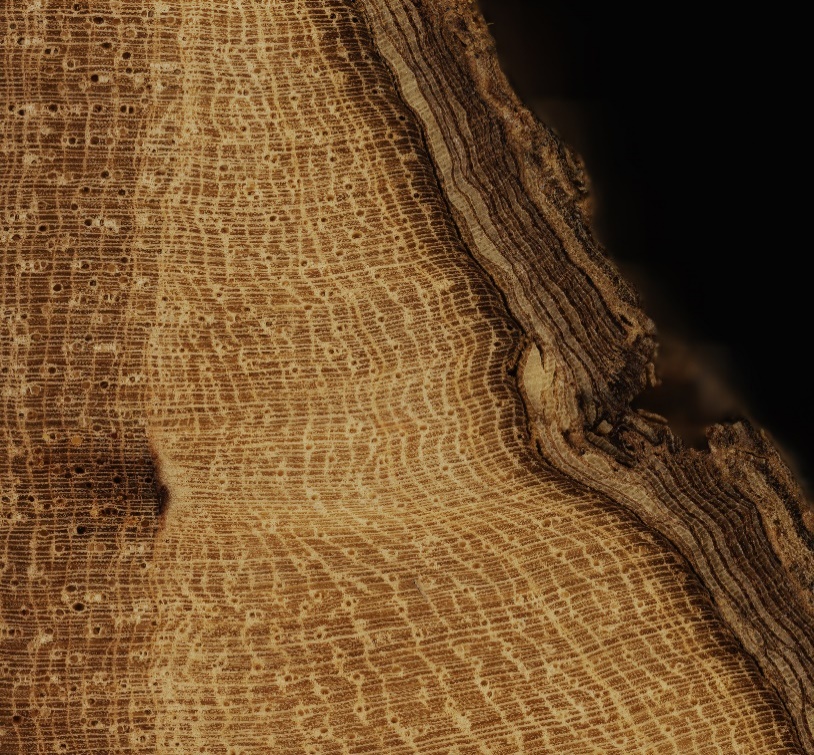  Tw68275 | 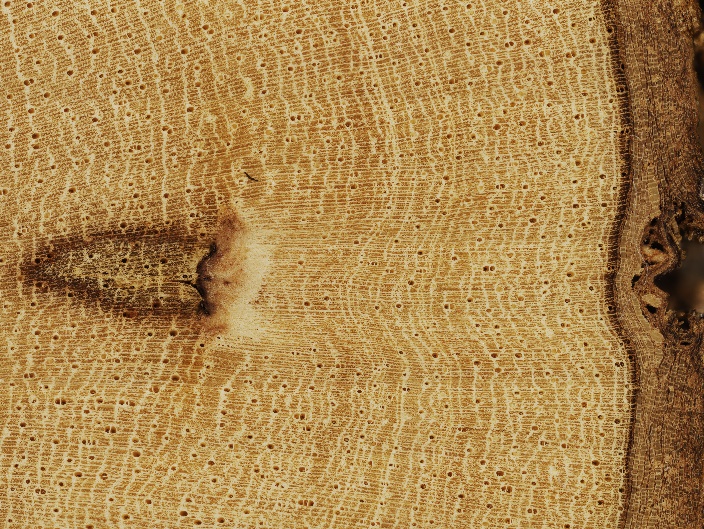  Tw69513 |
| --- | --- |

*Trilepisium madagascariense*

| 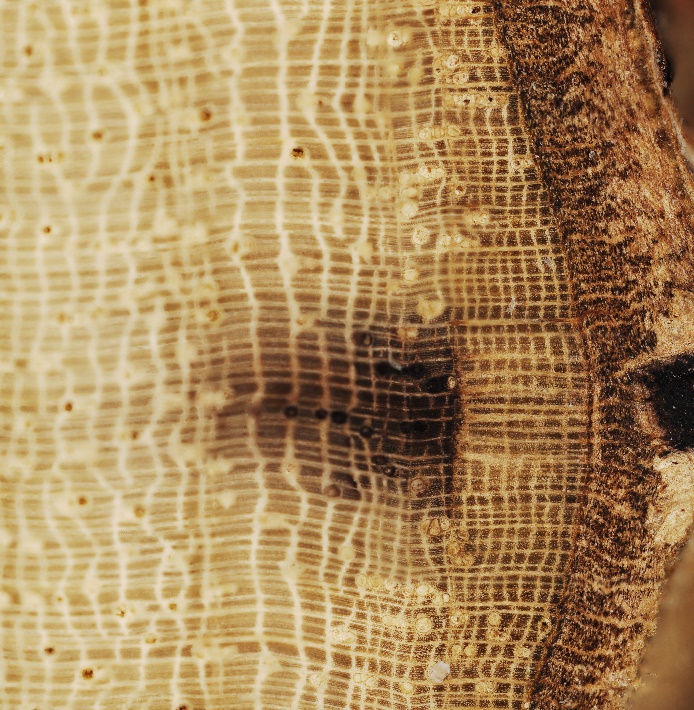  Tw68318 | 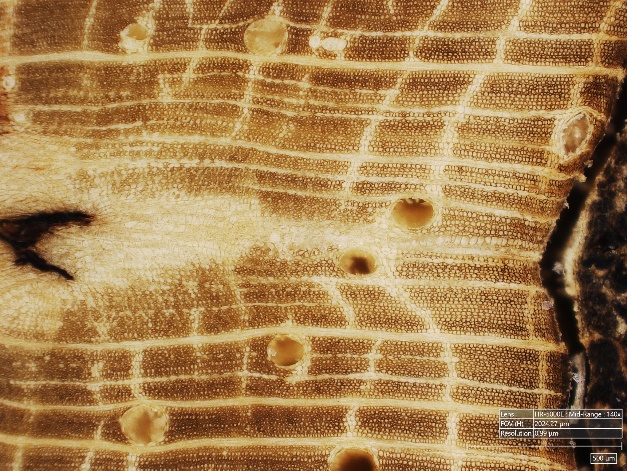  Tw69475 |
| --- | --- |
